# Supplementary figures and images for: Isolation, identification and antibacterial activity of endophytes from the seeds of Panax japonicus
Source: PLoS One. 2025 Oct 9;20(10):e0330436. doi: 10.1371/journal.pone.0330436 (PMC12510572; doi:10.1371/journal.pone.0330436)

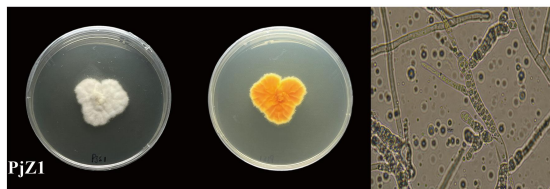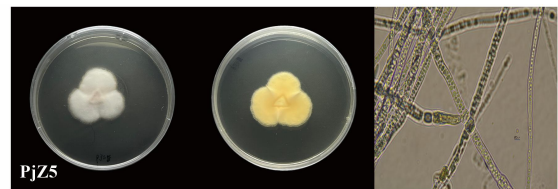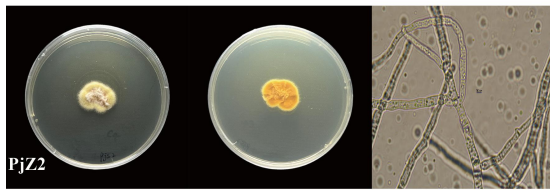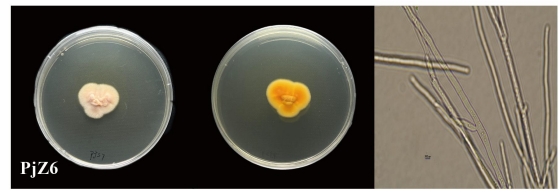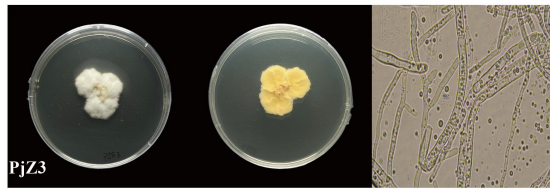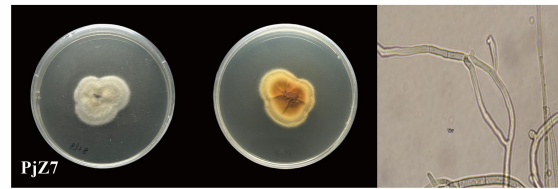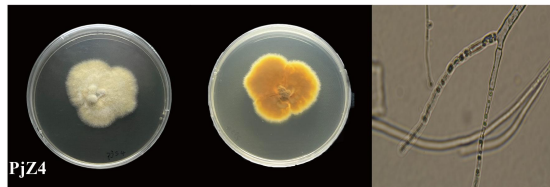

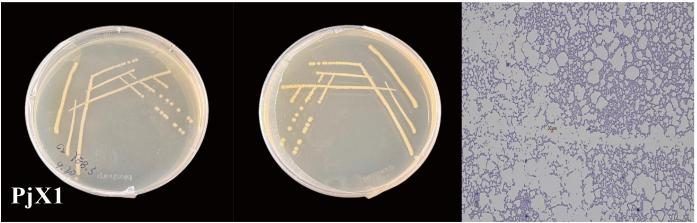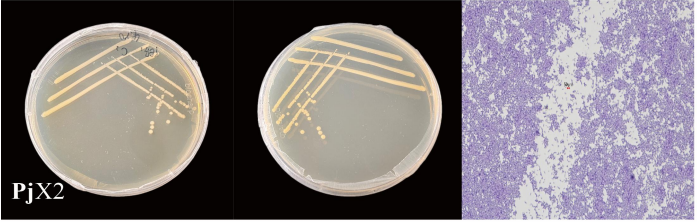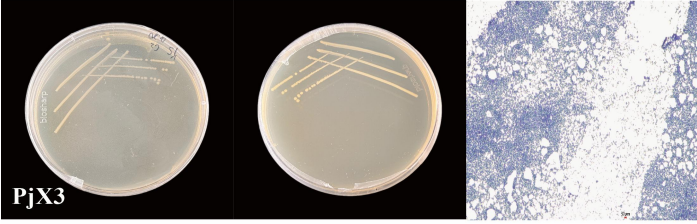

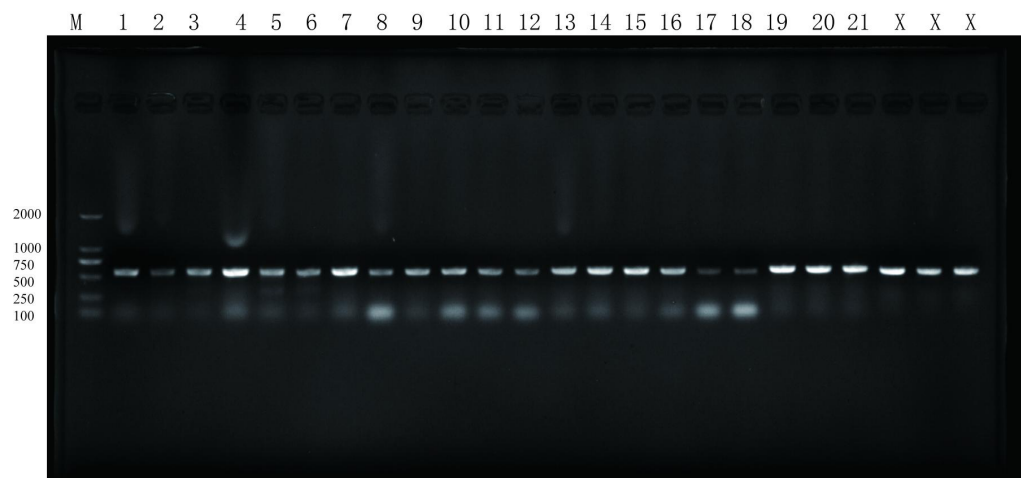

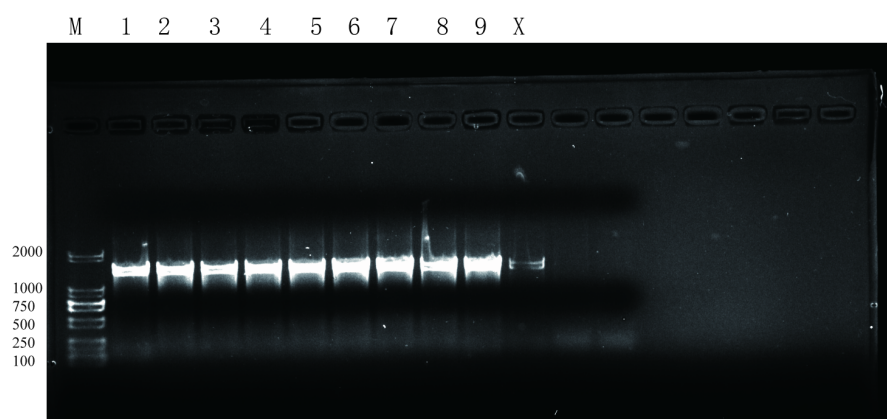

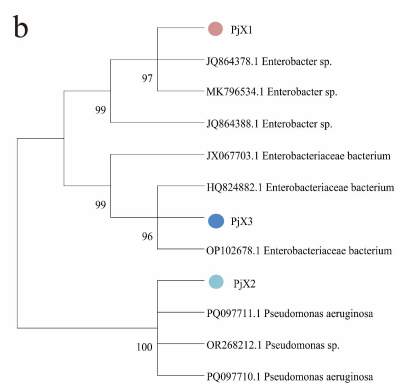

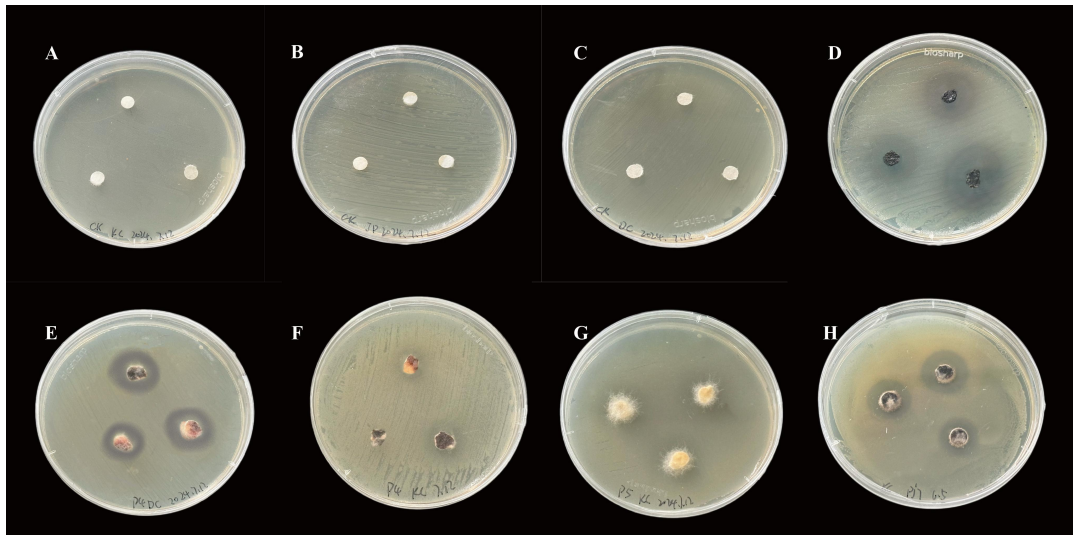

Supplement: S1 File — (PDF) [file pone.0330436.s001.pdf]
