# Supplementary material for: Isolation, identification and antibacterial activity of endophytes from the seeds of Panax japonicus
Source: PLoS One. 2025 Oct 9;20(10):e0330436. doi: 10.1371/journal.pone.0330436 (PMC12510572; doi:10.1371/journal.pone.0330436)
Supplement: Attachment — Submitted filename: PONE-D-24-46415_R1_reviewer1.pdf [file pone.0330436.s004.pdf]

PLOS ONE

Isolation, identification and antibacterial activity of endophyte from the seeds of Panax japonicus

--Manuscript Draft--

**Manuscript Number:**	PONE-D-24-46415R1
**Article Type:**	Research Article
**Full Title:**	Isolation, identification and antibacterial activity of endophyte from the seeds of Panax japonicus
**Short Title:**	Isolation, identification and antibacterial activity of endophytic bacteria from the seeds of Panax japonicus
**Corresponding Author:**	Lai Zhang<br>Anshun University<br>Guizhou Anshun, CHINA
**Keywords:**	Keywords:Panax japonicus; Seeds; Endophyte; Isolation and identification; Endophytic antibacterial activity
**Abstract:**	The purpose of this study is to isolate and identify endophytic bacteria from the seeds of Panax japonicus, finding a dominant strain that favors seed germination in Panax japonicus and to provide theoretical reference for further research on the effect of endophytic bacteria on seed germination. Methods: The endophytic bacteria were isolated from the seeds by plate separation method. The isolated endophytes were identified by morphology, ITS and 16S rDNA sequence analysis.The antibacterial activity of the isolated endophytes and their metabolites was studied by disk method. Results: The seed was disinfected with 75% alcohol for 30s and 1% HgCl2 for 9min, and the contamination rate was the lowest (3.33%). Seven endophytic fungi strains were isolated from the seeds of Panax japonicus, named PjZ1, PjZ2, PjZ3, PjZ4, PjZ5, PjZ6, and PjZ7, respectively. Four strains belonged to the genus Fusarium tricinctum, one to Fusarium reticulatum, one to Fusarium sarcochroum, and one to Alternaria alternata; Three strains of endophytic bacteria were isolated and named PjX1, PjX2 and PjX3, respectively. After morphological observation and 16S rDNA gene sequencing, two strains belong to the genus Enterobacteriaceae bacterium and one belongs to the genus Pseudomonas sp. Among the endophytic fungal strains isolated, only PjZ4 showed significant bacteriostatic activity against Escherichia coli, PjZ3, PjZ4, PjZ5 and PjZ7 had antibacterial activity against Bacillus subtilis, and none of the 7 strains had antibacterial activity against Staphylococcus aureus. It was found that the metabolites of 7 endophytic fungi and 3 endophytic bacteria had no antibacterial activity. Conclusion: In this study, we investigated the method of bamboo ginseng seed disinfection, and the results showed that 75% alcohol disinfection for 30s and 1% HgCl2 disinfection for 9min resulted in the lowest contamination rate of 3.33%, in which the greatest influence on the seed disinfection effect was the concentration of HgCl2. Meanwhile, 11 endophyte species of Panax japonicus seeds were isolated and identified, Fusarium spp. and Alternaria alternata. were not the dominant species in the growth and development process of Panax japonicus while Enterobacteriaceae and Pseudomonas sp. were the dominant species in the promotion of plant growth and development, and provides a theoretical reference for further research on the biological functions and active substances of endophytic fungi in the seeds of Panax japonicus.
**Order of Authors:**	Rui Jin
	Tingting Tang
	Xilun Huang
	Juan Huang
	E Liang
	Lai Zhang
**Response to Reviewers:**	Response to Reviewers<br>Dear editors and reviewers,

Thank you for your comments on the title of our manuscript 'Isolation, identification and antibacterial activity of endophyte from the seeds of Panax japonicus '. Your comments will help us to revise and improve the manuscript, and will also play an important guiding role in our future research in the Panax japonicus or other fields. We are very grateful to you for your comments and comments on the content of our manuscripts, especially for the questions we have pointed out in words. We have carefully studied these comments and tried to modify the manuscript, hoping to get your approval. Revisions are marked in red in the manuscript. The following is a response to your comments.

Editor

1.Please ensure that your manuscript meets PLOS ONE's style requirements, including thosefor file naming.

Response: Dear Editor, thank you for your reminder that we have modified the format and naming according to the journal requirements.

2.Please state what role the funders took in the study.

Response:Thanks to the editors for being so careful, we have clarified in the Cover letter: “The above four project funds provide financial support and project guidance for this research.”

3.We note that you have provided funding information that is currently declared in your Funding Statement. However, funding information should not appear in the Acknowledgments section or other areas of your manuscript. We will only publish funding information present in the Funding Statement section of the online submission form.Please remove any funding-related text from the manuscript and let us know how you would like to update your Funding Statement.

Response:Thanks for the editor's reminder and suggestion, we have made modifications, deleted the text related to funding from the manuscript, and included the funding information in the Cover letter.

4.When completing the data availability statement of the submission form, you indicated that you will make your data available on acceptance. We strongly recommend all authors decide on a data sharing plan before acceptance, as the process can be lengthy and hold up publication timelines. Please note that, though access restrictions are acceptable now, your entire data will need to be made freely accessible if your manuscript is accepted for publication. This policy applies to all data except where public deposition would breach compliance with the protocol approved by your research ethics board. If you are unable to adhere to our open data policy,please kindly revise your statement to explain your reasoning and we will seek the editor's input on an exemption. Please be assured that, once you have provided your new statement, the assessment of your exemption will not hold up the peer review process.

Response:Thanks for the editor's suggestion, we have started to prepare the work of data uploading and data sharing, and we promise to do our best to complete the work before the manuscript is accepted.

5.PLOS ONE now requires that authors provide the original uncropped and unadjusted images underlying all blot or gel results reported in a submission’s figures or Supporting Information Files.In your cover letter, please note whether your blot/gel image data are in Supporting Informationor posted at a public data repository, provide the repository URL if relevant, and provide specificdetails as to which raw blot/gel images, if any, are not available. Email us at plosone@plos.org if you have any questions.

Response:Thanks for the editors' valuable feedback and suggestions on the chart information. We have put the unadjusted chart into the file separately and explained it in the Cover letter: we will agree that blot/gel image data are in Supporting Information or posted at a public data repository.

6.Please remove your figures from within your manuscript file, leaving only the individual TIFF/EPS image files, uploaded separately ,and please include your tables as part of your main manuscript and remove the individual files.

Response:Thanks to the careful reminding of the editor, we have uploaded the single TIFF/EPS image file and deleted the chart from the manuscript, and the single table file has also been deleted.

Reviewer 1

Abstract (Lines 39-65):

• Line 40: Consider stating the specific aim of this study more clearly, such as: “The aim of this study was to isolate...”

Response:We agree with the reviewer on this point, and we have revised it in the

Powered by Editorial Manager® and ProduXion Manager® from Aries Systems Corporation

manuscript: “ The purpose of this study is to isolate and identify endophytic bacteria from the seeds of Panax japonicus, finding a dominant strain that favors seed germination in Panax japonicus and to provide theoretical reference for further research on the effect of endophytic bacteria on seed germination. ”

• Line 48: It is confusing to read "Seven endophytic fungi strains were isolated" immediately followed by mentioning that "PjZ1, PjZ3, PjZ4 and PjZ5 were identified as Fusarium". This makes it appear that only these four were fungi and not all 7 strains. You should rephrase this to clarify
that ALL 7 are fungal.

Response:We thank the reviewer for making this important point, which we have elaborated in the manuscript: “Seven endophytic fungi strains were isolated from the seeds of Panax japonicus, named PjZ1, PjZ2, PjZ3, PjZ4, PjZ5, PjZ6, and PjZ7, respectively. Four strains belonged to the genus Fusarium tricinctum, one to Fusarium reticulatum, one to Fusarium sarcochroum, and one to Alternaria alternata”

• Line 55: Instead of "Only PjZ4 had antibacterial activity", it may be more appropriate to say,"Among the isolates, only PjZ4 showed significant antibacterial activity...". This highlights that the other fungal isolates did have some inhibition on Bacillus subtilis, even if not significant.

Response:We thank the reviewer for pointing out a more appropriate statement for us, and we have readjusted it: “Among the endophytic fungal strains isolated, only PjZ4 showed significant bacteriostatic activity against Escherichia coli. ”

• Line 58: Conclusion: It is advised to have a stronger conclusion. For instance, the authors could summarize the main findings, including the genus level of the identified strains, and highlight the significance of their study for future research.

Response:Thanks to the reviewer for his valuable suggestions. In view of the suggestions, we have made a new explanation after careful consideration:“In this study, we investigated the method of bamboo ginseng seed disinfection, and the results showed that 75% alcohol disinfection for 30s and 1% $HgCl_2$ disinfection for 9min resulted in the lowest contamination rate of 3.33%, in which the greatest influence on the seed disinfection effect was the concentration of $HgCl_2$. Meanwhile, 11 endophyte species of Panax japonicus seeds were isolated and identified, Fusarium spp. and Alternaria alternata. were not the dominant species in the growth and development process of Panax japonicus while Enterobacteriaceae and Pseudomonas sp. were the dominant species in the promotion of plant growth and development, and provides a theoretical reference for further research on the biological functions and active substances of endophytic fungi in the seeds of Panax japonicus.”

• Line 63: The keywords could be improved by making them more specific. For example, replace "Antibacterial activity" with "Endophytic antibacterial activity".

Response:Thanks for your helpful suggestions, we have updated the keyword "Antibacterial activity" to "Endophytic antibacterial activity"

Introduction (Lines 66-81):

• Line 68: The phrase "also known as Disporopsis fuscopicta Hance" is an unnecessary detail here as it is not the same genus.

Response:We appreciate your reminder that we have removed the word from the manuscript.

• Line 70: Should add citations to support that "Pharmacological studies have shown that Panax japonicus..."

Response:We are grateful for your reminder that in the manuscript, our citation for this part reads [2-4]:[2]SHEN Peiyao, ZHANG Ye, LI Yuqin, YAN Jianye, WANG Yuanqing. Research progress on biological activity and quality control of Panacis Japonici Rhizoma[J]. Food and Machinery,2021,37(11):211-220. [3]GUO Zhe, FENG Zhitao, ZHANG Haoran, YAN Lan, LIANG Mingge, MEI Zhigang, CAI Sanjin. Progress of research on Panax japonicus and its preparation for the treatment of rheumatoid arthritis[J]. Chinese Materia Medica,2019,42(04):941-944. [4]LI Chunyan, ZHANG Jie, LI Jinping, et al. Research on Chemical Constituents and Biological Activities of Rhizoma of Panax japonicus[J]. Introduction to Traditional Chinese Medicine,2012,18(04):68-71.DOI:10.13862/j.cnki.cn43-1446/r.2012.04.050.

• Line 75: The statement regarding lack of reports on endophytic fungi/bacteria from seeds is good, but there needs to be something to indicate why this is valuable. For example, why are seeds and what importance is this to P. japonicus? And Line 77-80: The end of the introduction, there should be something to emphasize the biological functions of the bacteria isolated.

Response:We would like to thank the reviewer for pointing out this problem. The clarification of this problem will make the purpose of this paper more clear. Therefore,

Powered by Editorial Manager® and ProduXion Manager® from Aries Systems Corporation

we have further elaborated this part in the manuscript:“Because of the high efficacy and high price of the rare wild Chinese herbal medicine, it is almost difficult to find wild resources of Panax japonicus in the wild, so the artificial propagation of Panax japonicus seeds is urgent.Therefore, the selection of Panax japonicus seeds, leaves and stems for seedling propagation is a better choice of explants. It is noteworthy that the seeds of Panax japonicus have the phenomenon of after-ripening, and the contamination rate is high during the germination process of histoculture, which makes the germination difficult. Therefore, we carried out further exploration. It has been shown that endophytes of medicinal plants are important for promoting the growth of medicinal plants, improving the quality of medicinal plants, and eliminating the barriers of continuous cultivation.”

Materials and Methods (Lines 82-162):

• Line 86: Provide the geographic coordinates or location details of the experimental site.

Response:We would like to thank the reviewer for suggesting that our detailed address is"Guizhou Innovation Center of Mountain Specialty and Efficient Agriculture" of Anshun College, Anshun City, Guizhou Province, China, has been presented in the manuscript.

• Line 90: The composition of NA (Nutrient Agar) should be described better. The recipe is given as "Peptone 10g, Beef Paste 3g, Sodium Chloride 5g, Agar 20g, distilled water 1000mL, pH:7.2." It would be better practice to say "Beef Extract" instead of "Beef Paste", as well as identify if peptone is tryptone or another form.

Response:We would like to thank the reviewers for their careful comments on this and we have corrected "Beef Extract" to" Beef paste".

• Line 103: Provide a citation or additional details to justify the use of this orthogonal testing method.

Response:Thank you very much for your professional advice and we have added relevant citations in this part of the manuscript:[8]ZHU Lili,ZHANG Yemeng,LI Wancai,et al. Comprehensive evaluation of the effects of different disinfectants on surface disinfection and germination of quinoa seeds[J]. Journal of Biology,2023,40(4):83-89. DOI:10.3969/j.issn.2095-1736.2023.04.083.

• Line 107-109: It is difficult to follow that section. Re-word and clarify that after disinfection, the seeds were plated to analyze contamination rate before proceeding with isolation.

Response:We thank the reviewers for their important comments on this, which we have rephrased in the manuscript:“Prior to the isolation of endophytes from the seeds of Panax japonicus, the seeds of Panax japonicus were sterilized under different conditions and the contamination rate under different sterilization conditions were analyzed.”

• Line 117: Clarify why the seed is cut in half before plating.

Response:Thank you very much for your professional advice and we have added relevant citations in this part of the manuscript:[9]ZHANG Yubai, WEN Xiangshui, WANG Yumeng, et al. Isolation and characterization of endophytic fungi from the seeds of Fructus schisandrae chinensis and their fungicidal properties[J]. Chinese Materia Medica,2020,43(5):1087-1091. DOI:10.13863/j.issn1001-4454.2020.05.008.

• Line 119: What is meant by "seed preservation" here? It is better to say for "preservation of the isolated strains"

Response:We thank the reviewers for their very meticulous comments, which we have reorganized: “After the growth of mycelium, pick the mycelium for repeated isolation and purification for 2-3 times for preservation of the isolated strains.”

• Line 127-136: The morphological observation section lacks clarity. How many samples were observed per isolate? Were observations done in replicates? More detail on how results were "recorded" should be included.

Response:Thanks to the reviewer's constructive comments on this, we have re-elaborated in the manuscript: “Pick the purified fungal hyphae and make them into clinical slices, observe and record (hyphae color, hyphae morphology, spore morphology, etc.) through an optical microscope, and refer to "Fungal Identification Manual".etc. for preliminary identification of fungi. (Three strains on each medium for each strain and three strains on each medium for observation and identification).”

• Line 137-139: The DNA extraction procedure needs to be described with more detail: What is the kit that was used? Also, it is better to use "according to the manufacture's instructions" rather than just stating "according to the instructions".

Response: Thanks to the reviewer for alerting us to this problem, which we have reflected in the manuscript:“Endophytic fungal DNA was extracted according to the

	instructions of the fungal DNA extraction kit (Solarbio), and endophytic bacterial DNA was extracted according to the instructions of the bacterial DNA extraction kit. Subsequently, PCR amplification was performed using the DNA of each endophytic bacterium as a template, respectively."<br>• Line 144: The reaction volume and composition is confusing. It would be better to say "a 50uL reaction volume containing..." instead.<br>Response: Thanks to the reviewer's attention to the following details, we have corrected the statement:"Endophytic fungi were amplified by PCR using the universal primers of fungi, ITS1 (5'-TCCGTAGGTGAACCTGCGG-3') and ITS4 (5'-TCCTCCGCTTATTGATATGC-3') as primers, and the total volume of the PCR reaction system was 50μL, containing:4μL of DNA template, 2μL of each of ITS1 and ITS4, and 2×MasterMix 25μL, and add ddH2O 17μL."<br>• Line 151: The statement about the primer name is inconsistent, the previous section mentioned primer ITS1 and ITS4 but now are described as "primers 27F (5'-AGAGTTTGATCCTGGCTCAG-3') and 1492R (5'-TACGGCTACCTTGTTACGAC".<br>Response: Thanks to the reviewer for raising this question. The inconsistent names of primers are due to the fact that the endophytic bacteria isolated in our paper include fungi and bacteria, and the common primers of the two bacteria are different, so the names and sequences of the two primers appear in the paper.<br>• Line 157: What percentage of agarose gel was used in gel electrophoresis?<br>Response:Thanks for the reviewer's attention to this issue: In the manuscript we mentioned that the percentage of agarose gel used was 1%.<br>• Line 166: Please add more detail about the selection of pure isolates. For instance, whether single colonies were used to prepare the pure isolates,and Line 167: What does "adjusted to OD600=0.2 in 0.85% saline"means? Also, how was the 0.2 OD reading achieved?<br>Response:Thanks to the reviewers, we have described the process more clearly in the manuscript and updated the text:"The pure indicator strains (Staphylococcus aureus, Bacillus subtilis, Escherichia coli) were picked and inoculated onto LB medium by plate streaking method for overnight cultivation; Pick single colonies on LB medium again for plate streaking overnight culture; pick single colonies to 0.85% saline to prepare the bacterial solution, adjust the concentration of the bacterial solution and measured by UV-visible spectrophotometer (UV-5500, Shanghai, China), until OD600 = 0.2 ,and the bacterial solution was dipped into cotton swabs to evenly spread onto NA medium to make the bacterial plate containing the indicator strains."<br>• Line 174: It is good to mention the preparation of fungal cake, but describe the type of agar that was used,and Line 178: Please mention which drug-sensitive paper was used.<br>Response:Thanks to the reviewer for asking this question, we have revised it in the manuscript:"a perforator with a diameter of 0.7cm was used to prepare bacterial cakes on the endophytic fungal colonies cultivated for 10 d. The bacterial cakes were placed on the bacterial plate containing the indicator strains in 3 groups of replicates per endophytic fungal strain with blanks. The fungus cake was prepared on the endophytic fungal colonies with a 0.7 cm diameter punch, and placed on the fungus plate containing the indicator species, and 3 cakes were inoculated equidistant from each plate; 3 groups of replicates were made for each endophytic fungus, and the no antibiotics blank drug-sensitive paper(Shandong Best Microbial Technology Co., Ltd.) was used as the blank control, and the culture was incubated at a constant temperature of 28 ℃, and the bacterial inhibitory activity was observed after 3 d. The results were summarized as follows,the size of the ring of inhibition was recorded and the average value was calculated."<br>Results (Lines 163-205):<br>• Line 222-229: It would be better to include a table of the Gram staining morphology results forbetter data presentation.<br>Response:Thanks to the reviewer's suggestion, we have taken your suggestion and have added the table to the document:"Table 4 Morphology and Gram staining of endophytic bacteria in the seeds of Panax japonicus."<br>• Line 242: Avoid the use of the abbreviation for "16S ribosomal DNA", write as "16S rDNA" for consistency.<br>Response:Thank you very much for reviewing our manuscript so carefully. We have made consistent revisions in the manuscript.<br>• Line 242-261: The method of identifying the endophytes is unclear. You should state the database that was used, such as NCBI (National Center for Biotechnology Information).Also, it would help to give more details regarding the process of

Powered by Editorial Manager® and ProduXion Manager® from Aries Systems Corporation

phylogenetic tree construction.
Response:Thank you very much for the reviewer's careful suggestions, and we have taken the suggestions to supplement the manuscript:“BLAST search was performed at NCBI and a Utilization of the software MEGA11 phylogenetic tree was constructed (Fig.5), which revealed that the sequence similarity between endophytic fungus PjZ1 and accession number JX827458.1 was 100% and identified as Fusarium tricinctum;PjZ2 and accession number MT601889.1 had 100% sequence similarity and identified as Fusarium reticulatum.”
• Line 276: Clarify that “Antagonistic experiments” are referring to the testing of antibacterial activity. Also, clarify that the bacteria used in the antagonistic experiments were pathogenic.
Response:Thank you very much for the reviewer's suggestion. We have revised "Antagonistic experiments" to "Determination of bacteriostatic activity".
• Line 281-284: Describe the statistical significance of the differences observed and Line 298-307: The lack of activity should be presented in a table.
Response:Thank you very much for reminding us of this problem, we have added the table in the manuscript:Table 7 Inhibitory activity of endophytic fungal metabolites from the seeds of Panax japonicus and Table 8 Inhibitory activity of endophytic fungal metabolites from the seeds of Panax japonicus.
Discussion (Lines 308-376):
• Line 313: Clarify why the presence of Fusarium and Alternaria is important to discuss.
Response:Many thanks to the reviewer for raising this question, and we have made a new explanation in the manuscript:“Therefore, it is likely that the difficulty in seed germination of Panax japonicus is due to the action of these two endophytic fungi, and further research is needed to find antibiotics to inhibit the growth of this endophytic fungus in order to solve the problem of the difficulty in seed germination of Panax japonicus.”
• Line 315: Is this significant? What do the non-inhibitory results suggest?•Line 320: Again, why is it important to note the dominant fungi were not found?Line 335: The authors should further discuss the importance of their finding in P. japonicus.?Line 337-344: Needs a better transition to the importance of Pseudomonas and Enterobacteriaceae.Line 361-365: Reiterate more clearly. The authors should briefly list the identified species, but also highlight the dominant species in the study,and Line 366-376: The conclusion is not comprehensive enough and should be revised to clearly state the main outcomes, limitations, and implications for future studies.
Response:“The or thogonal experiment screened out the optimal disinfection conditions of Panax japonicus seeds as 75% alcohol disinfection for 30s and 1% $HgCl_2$ disinfection for 9min, which had the lowest contamination rate of 3.33%. Seven strains of endophytic fungi PjZ1, PjZ2, PjZ3, PjZ4, PjZ5, PjZ6, PjZ7 and three strains of endophytic bacteria PjX1, PjX2, PjX3 were isolated and screened from the seeds of Panax japonicus, and the strains of PjZ1, PjZ3, PjZ4, PjZ5 were identified as the fungi of the Fusarium genus, and the strains of PjZ2 and PjZ4 were identified as the fungi of the Fusarium genus. Fusarium reticulatum, PjZ6 is Fusarium sarcochroum, PjZ7 is Alternaria alternata; PjX1 and PjX3 are Enterobacteriaceae bacterium, and PjX2 is Pseudomonas aeruginosa. The results of inhibition experiments showed that only PjZ4 had inhibitory activity against E. coli, PjZ3, PjZ4, PjZ5, PjZ7 had inhibitory activity against Bacillus subtilis, and none of the 7 endophytic fungal strains had inhibitory activity against Staphylococcus aureus, and the metabolites of the 10 endophytic strains of endophytic fungi were antagonized with E. coli, Staphylococcus aureus, and Bacillus subtilis. The experiment showed that the metabolites of 10 strains of endophytic bacteria had no bacteriostatic activity against Escherichia coli, Staphylococcus aureus and Bacillus subtilis. After preliminary judgment, the isolated Fusarium spp. and Alternaria alternata. are not the dominant species in the growth and development process of Panax japonicus, and it can be inferred that the difficulty in seed germination of bamboo ginseng may be caused by the growth of these two genera of fungi inhibiting the synthesis of growth hormone by the seeds during the seed germination process. The isolated Enterobacteriaceae bacterium and Pseudomonas spp. are the dominant species in promoting plant growth and development, so these two genera of bacteria can be utilized in the cultivation process of Panax japonicus to promote its growth and development.The endophytes isolated in this study provide a theoretical basis for the screening of endophyte antagonist strains of Panax japonicus and for seed germination and disease control of Panax japonicus.”
Reviewer 1
For example, the paper's title is “Isolation, identification and antibacterial activity of

Powered by Editorial Manager® and ProduXion Manager® from Aries Systems Corporation

	endophytic bacteria from the seeds of Panax japonicus." However, the MS reports both bacterial and fungal endophytes. For instance, under the abstract, the authors write, “Seven endophytic fungi strains were isolated from the seeds of Panax japonicus, named…………”<br>Response:Thanks to the reviewer for raising this question, we have revised the title into a more accurate text.<br>Again, thank you for giving us the opportunity to strengthen our manuscript with your valuable comments and queries. We have worked hard to incorporate your feedback and hope that these revisions persuade you to accept our submission.<br>Best regards.<br>Yours sincerely, Rui Jin<br>Corresponding author: Name: Lai Zhang<br>E-mail: 975575681@qq.com<br>January 14, 2025
**Additional Information:**	
**Question**	**Response**
**Financial Disclosure**<br><br>Enter a financial disclosure statement that describes the sources of funding for the work included in this submission. Review the submission guidelines for detailed requirements. View published research articles from *PLOS ONE* for specific examples.<br><br>This statement is required for submission and **will appear in the published article** if the submission is accepted. Please make sure it is accurate.<br><br>**Funded studies**<br>Enter a statement with the following details:<br>• Initials of the authors who received each award<br>• Grant numbers awarded to each author<br>• The full name of each funder<br>• URL of each funder website<br>• Did the sponsors or funders play any role in the study design, data collection and analysis, decision to publish, or preparation of the manuscript?<br><br>Did you receive funding for this work?	Yes
Please add funding details.<br>as follow-up to "**Financial Disclosure**<br><br>Enter a financial disclosure statement that describes the sources of funding for the work included in this submission. Review	Science and Technology Funded Project of Guizhou Provincial Science and Technology Department (Guizhou Science and Technology Foundation -ZK Grant NO.(2023) Key 002).<br>National Natural Science Foundation of China (Grant NO.31660252).<br>Guizhou Provincial Department of Education Youth Science and Technology Talent Development Project (Qian Jiao He KY character Grant NO.[2022]029).

the submission guidelines for detailed requirements. View published research articles from *PLOS ONE* for specific examples.<br><br>This statement is required for submission and **will appear in the published article** if the submission is accepted. Please make sure it is accurate.<br><br>**Funded studies**<br>Enter a statement with the following details:<br>• Initials of the authors who received each award<br>• Grant numbers awarded to each author<br>• The full name of each funder<br>• URL of each funder website<br>• Did the sponsors or funders play any role in the study design, data collection and analysis, decision to publish, or preparation of the manuscript?<br><br>Did you receive funding for this work?"	Anshun College graduate Research Innovation project asxyyjscx Grant NO.202410. The funders had no role in study design, data collection and analysis, decision to publish, or preparation of the manuscript.
Please select the country of your main research funder (please select carefully as in some cases this is used in fee calculation).<br>as follow-up to "**Financial Disclosure**<br><br>Enter a financial disclosure statement that describes the sources of funding for the work included in this submission. Review the submission guidelines for detailed requirements. View published research articles from *PLOS ONE* for specific examples.<br><br>This statement is required for submission and **will appear in the published article** if the submission is accepted. Please make sure it is accurate.	CHINA - CN

**Funded studies**<br>Enter a statement with the following details:<br>• Initials of the authors who received each award<br>• Grant numbers awarded to each author<br>• The full name of each funder<br>• URL of each funder website<br>• Did the sponsors or funders play any role in the study design, data collection and analysis, decision to publish, or preparation of the manuscript?<br><br>Did you receive funding for this work?"	
**Competing Interests**<br><br>Use the instructions below to enter a competing interest statement for this submission. On behalf of all authors, disclose any competing interests that could be perceived to bias this work—acknowledging all financial support and any other relevant financial or non-financial competing interests.<br><br>This statement is required for submission and **will appear in the published article** if the submission is accepted. Please make sure it is accurate and that any funding sources listed in your Funding Information later in the submission form are also declared in your Financial Disclosure statement.<br><br>View published research articles from *PLOS ONE* for specific examples.	I have read the journal's policy and the authors of this manuscript have the following competing interests:No conflict of interest exits in the submission of this manuscript, and manuscript is approved by all authors for publication.I would like to declare on behalf of my co-authors that the work described was original research that has not been published previously, and not under consideration for publication elsewhere, in whole or in part. All the authors listed have approved the manuscript that is enclosed.

Powered by Editorial Manager® and ProduXion Manager® from Aries Systems Corporation

**NO authors have competing interests**<br><br>Enter: *The authors have declared that no competing interests exist.*<br><br>**Authors with competing interests**<br><br>Enter competing interest details beginning with this statement:<br><br>*I have read the journal's policy and the authors of this manuscript have the following competing interests: [insert competing interests here]*<br><br>* typeset	
**Ethics Statement**<br><br>Enter an ethics statement for this submission. This statement is required if the study involved:<br><br>• Human participants<br>• Human specimens or tissue<br>• Vertebrate animals or cephalopods<br>• Vertebrate embryos or tissues<br>• Field research<br><br>Write "N/A" if the submission does not require an ethics statement.<br><br>General guidance is provided below. Consult the submission guidelines for detailed instructions. **Make sure that all information entered here is included in the Methods section of the manuscript.**	N/A

Powered by Editorial Manager® and ProduXion Manager® from Aries Systems Corporation

**Format for specific study types**<br><br>**Human Subject Research (involving human participants and/or tissue)**<br>• Give the name of the institutional review board or ethics committee that approved the study<br>• Include the approval number and/or a statement indicating approval of this research<br>• Indicate the form of consent obtained (written/oral) or the reason that consent was not obtained (e.g. the data were analyzed anonymously)<br><br>**Animal Research (involving vertebrate animals, embryos or tissues)**<br>• Provide the name of the Institutional Animal Care and Use Committee (IACUC) or other relevant ethics board that reviewed the study protocol, and indicate whether they approved this research or granted a formal waiver of ethical approval<br>• Include an approval number if one was obtained<br>• If the study involved *non-human primates*, add *additional details* about animal welfare and steps taken to ameliorate suffering<br>• If anesthesia, euthanasia, or any kind of animal sacrifice is part of the study, include briefly which substances and/or methods were applied<br><br>**Field Research**<br><br>Include the following details if this study involves the collection of plant, animal, or other materials from a natural setting:<br>• Field permit number<br>• Name of the institution or relevant body that granted permission	
**Data Availability**<br><br>Authors are required to make all data underlying the findings described fully available, without restriction, and from the time of publication. PLOS allows rare exceptions to address legal and ethical concerns. See the PLOS Data Policy and FAQ for detailed information.	Yes - all data are fully available without restriction

A Data Availability Statement describing where the data can be found is required at submission. Your answers to this question constitute the Data Availability Statement and **will be published in the article**, if accepted.<br><br>**Important:** Stating 'data available on request from the author' is not sufficient. If your data are only available upon request, select 'No' for the first question and explain your exceptional situation in the text box.<br><br>Do the authors confirm that all data underlying the findings described in their manuscript are fully available without restriction?	
**Describe where the data may be found in full sentences. If you are copying our sample text, replace any instances of XXX with the appropriate details.**<br><br>• If the data are **held or will be held in a public repository**, include URLs, accession numbers or DOIs. If this information will only be available after acceptance, indicate this by ticking the box below. For example: *All XXX files are available from the XXX database (accession number(s) XXX, XXX.*).<br>• If the data are all contained **within the manuscript and/or Supporting Information files**, enter the following: *All relevant data are within the manuscript and its Supporting Information files.*<br>• If neither of these applies but you are able to provide **details of access elsewhere**, with or without limitations, please do so. For example:<br><br>*Data cannot be shared publicly because of [XXX]. Data are available from the XXX Institutional Data Access / Ethics Committee (contact via XXX) for researchers who meet the criteria for access to confidential data.*<br><br>*The data underlying the results presented in the study are available from (include the name of the third party*	All relevant data are within the manuscript and its Supporting Information files

and contact information or URL).<br>• This text is appropriate if the data are owned by a third party and authors do not have permission to share the data.<br><br>* typeset	
Additional data availability information:	Tick here if the URLs/accession numbers/DOIs will be available only after acceptance of the manuscript for publication so that we can ensure their inclusion before publication.

Powered by Editorial Manager® and ProduXion Manager® from Aries Systems Corporation

Click here to access/download;Manuscript;Manuscript.docx

Isolation, identification and antibacterial activity of endophyte from the seeds of *Panax japonicus*

Rui Jin [1,2*] ,Tingting Tang [1,2*] , Xilun Huang [1,2] , Juan Huang [2] , E Liang [2] , Lai Zhang [2*]

[1]School of Life Science, Guizhou Normal University,Guizhou,Guiyang,China;

[2]Anshun university/Innovation Center for Efficient Agricultural of Guizhou Mountain Characteristics,Guizhou,Anshun,China

*Corresponding author

E-mail:975575681@qq.com.(LZ)

Author contribution statement:

Rui Jin(first author): Determine experimental steps, conduct experiments, software analysis, writing - manuscript;

Tingting Tang(co-first author) : Data collation, writing - original manuscript;

Xilun Huang: Image collation and analysis, sequence analysis;

Juan Huang: Software application and result verification

E Liang: Writing - reviewing and editing;

Lai Zhang(corresponding author): Conceptualization, funding acquisition, resources,

supervision, article revision and review.

Abstract

The purpose of this study is to isolate and identify endophytic bacteria from the seeds of *Panax japonicus*, finding a dominant strain that favors seed germination in *Panax japonicus* and to provide theoretical reference for further research on the effect of endophytic bacteria on seed germination. **Methods:** The endophytic bacteria were isolated from the seeds by plate separation method. The isolated endophytes were identified by morphology, ITS and 16S rDNA sequence analysis.The antibacterial activity of the isolated endophytes and their metabolites was studied by disk method. **Results:** The seed was disinfected with 75% alcohol for 30s and 1% $HgCl_2$ for 9min, and the contamination rate was the lowest (3.33%). Seven endophytic fungi strains were isolated from the seeds of *Panax japonicus*, named *Pj*Z1, *Pj*Z2, *Pj*Z3, *Pj*Z4, *Pj*Z5, *Pj*Z6, and *Pj*Z7, respectively. Four strains belonged to the genus *Fusarium tricinctum,* one to *Fusarium reticulatum,* one to *Fusarium sarcochroum,* and one to *Alternaria alternata*; Three strains of endophytic bacteria were isolated and named *Pj*X1, *Pj*X2 and *Pj*X3, respectively. After morphological observation and 16S rDNA gene sequencing, two strains belong to the genus *Enterobacteriaceae bacterium* and one belongs to the genus *Pseudomonas sp*. Among the endophytic fungal strains isolated, only *Pj*Z4 showed significant bacteriostatic activity against *Escherichia coli*, *Pj*Z3, *Pj*Z4, *Pj*Z5 and *Pj*Z7 had antibacterial activity against Bacillus subtilis, and none of the 7 strains had antibacterial activity against Staphylococcus aureus. It was found that the metabolites of 7 endophytic fungi and 3 endophytic bacteria had no antibacterial activity. **Conclusion:** In this study, we investigated the method of bamboo ginseng seed disinfection, and the results showed that 75% alcohol disinfection for 30s and 1% $HgCl_2$ disinfection for 9min resulted in the lowest contamination rate of 3.33%, in which the greatest influence on the seed disinfection effect was the concentration of $HgCl_2$. Meanwhile, 11 endophyte species of *Panax japonicus* seeds were isolated and identified, *Fusarium spp*. and *Alternaria alternata*. were not the dominant species in the growth and development process of *Panax japonicus* while *Enterobacteriaceae* and *Pseudomonas sp.* were the dominant species in the promotion of plant growth and

development, and provides a theoretical reference for further research on the biological functions and active substances of endophytic fungi in the seeds of *Panax japonicus*.

Keywords:*Panax japonicus*; Seeds; Endophyte; Isolation and identification; Endophytic antibacterial activity

Itroduction

Panax japonicus is the dried rhizome of *Panax japonicus* C.A. Mey..The chemical composition mainly includes saponins, sugars, amino acids, volatile oils, flavonoids, nucleosides and inorganic salts, etc.[1].Pharmacological studies have shown that *Panax japonicus* has a good therapeutic effects in enhancing immunity, anti-fatigue, cardioprotection, anti-tumor, anti-myocardial ischemia, sedation and analgesia, and treatment of rheumatoid arthritis[2-4]. Because of the high efficacy and high price of the rare wild Chinese herbal medicine, it is almost difficult to find wild resources of *Panax japonicus* in the wild, so the artificial propagation of *Panax japonicus* seeds is urgent[5].Therefore, the selection of *Panax japonicus* seeds, leaves and stems for seedling propagation is a better choice of explants. It is noteworthy that the seeds of *Panax japonicus* have the phenomenon of after-ripening, and the contamination rate is high during the germination process of histoculture, which makes the germination difficult. Therefore, we carried out further exploration. It has been shown that endophytes of medicinal plants are important for promoting the growth of medicinal plants, improving the quality of medicinal plants, and eliminating the barriers of continuous cultivation[6].Scholars have isolated endophytic bacteria from roots, stems, leaves, fruits,and rhizomes of *Panax japonicus*[7].However, the isolation of endophytic fungi and bacteria from the seeds of *Panax japonicus* has not yet been reported. Therefore, the isolation of endophytic bacteria from the seeds of *Panax japonicus* and their classification, characterization,and bacteriostatic studies can provide certain theoretical references for the further development of the research on the biological functions and active substances of the endophytic bacteria in the seeds of *Panax japonicus*, as well as for the germination of the seeds of *Panax japonicus.*

Materials and Methods

Plant material

Fresh seeds of *Panax japonicus* were obtained from the experimental site of *Panax japonicus* cultivation in the "Guizhou Innovation Center of Mountain Specialty and Efficient Agriculture" of Anshun College, Anshun City, Guizhou Province, China.

Culture medium

Fungal isolation medium: Potato Dextrose Agar Medium (PDA solid medium) 40.1g, distilled water 1000mL, pH:5.8. Bacterial isolation medium: Beef Extract Peptone Medium (NA solid medium): Peptone 10g, Beef Extract 3g, Sodium Chloride 5g, Agar 20g, distilled water 1000mL, pH:7.2. Experimental Medium for Fungal Inhibition Nutrient broth (NB liquid medium): peptone 10g, Beef Extract 3g, sodium chloride 5g, distilled water 1000mL, pH: 7.2, Beef Extract Peptone Medium (NA solid medium). Fungal fermentation medium: PDB liquid medium, Beef Extract Peptone Medium (NA solid medium), nutrient broth (NB liquid medium). Bacterial fermentation medium: LB solid medium, LB liquid medium, MH medium.

Bacterial strain

Strains Staphylococcus aureus, Escherichia coli, Bacillus subtilis, purchased from Guangdong Huankai Biotechnology Co.

Screening of seed disinfection conditions

With 75% alcohol disinfection time of 30s, 60s, 90s, $HgCl_2$ concentration of 0.1%, 0.5%, 1.0%, $HgCl_2$ disinfection time of 6min, 12min, 18min as the influencing factors, a 3-factor, 3-level orthogonal test was designed according to the orthogonal table of L_9 (3^3), with a total of 9 treatments[8].The results of the experiment were analyzed using the software SPSS.

Disinfection

Prior to the isolation of endophytes from the seeds of *Panax japonicus*, the seeds

of *Panax japonicus* were sterilized under different conditions and the contamination rate under different sterilization conditions were analyzed.The seeds were rinsed under running water and then sterilized with the above orthogonal design combinations.After disinfection, the seeds were inoculated on MS medium and the contamination rate was counted.

Isolation and purification of endophytic fungi

Take the fresh seeds of *Panax japonicus* and wash them under running water, then rinse them 1-3 times with sterile water, disinfect them with 75% alcohol for 30s, 1% $HgCl_2$ for 9min, rinse them with sterile water for 2-3 times and then dry them with sterile filter paper of 5 cm^2 in size, peel them and then cut them in half, put them on PDA medium with the cut side facing down[9], and then place them in an incubator at 28℃ for cultivation. After the growth of mycelium, pick the mycelium for repeated isolation and purification for 2-3 times for preservation of the isolated strains.

Isolation and purification of endophytic bacteria

Take fresh seeds of *Panax japonicus* and rinse them under running water, then rinse them 1-3 times with sterile water, the same as 1.6 Seed Disinfection Method, peel the seeds and cut them in half, place them on NA medium with the cut side facing down, and place them in the incubator at 28℃ for cultivation. After the growth of colonies, pick the strains and line the purification, purify 2-3 times to keep the seeds.

Identification of endophytes

Morphological observation

Pick the purified fungal hyphae and make them into clinical slices, observe and record (hyphae color, hyphae morphology, spore morphology, etc.) through an optical microscope, and refer to "Fungal Identification Manual".[10] etc. for preliminary identification of fungi. (Three strains on each medium for each strain and three strains on each medium for observation and identification).The purified bacterial strains were

subjected to continuous observation, and records were made (diameter size, color, morphology, dryness, whether transparent or not, etc. of the colonies). Pick a small amount of bacterial organisms to make clinical slices for Gram staining, and refer to the "Manual of Identification of Common Bacterial Systems" for preliminary analysis and identification[11].Make preliminary analysis and identification.

Molecular biology identification: Endophytic fungal DNA was extracted according to the instructions of the fungal DNA extraction kit (Solarbio), and endophytic bacterial DNA was extracted according to the instructions of the bacterial DNA extraction kit. Subsequently, PCR amplification was performed using the DNA of each endophytic bacterium as a template, respectively.

Endophytic fungi were amplified by PCR using the universal primers of fungi, ITS1 (5'-TCCGTAGGTGAACCTGCGG-3') and ITS4 (5'-TCCTCCGCTTATTGATATGC-3') as primers, and the total volume of the PCR reaction system was 50μL, containing:4μL of DNA template, 2μL of each of ITS1 and ITS4, and 2×MasterMix 25μL, and add ddH_2O 17μL. PCR reaction conditions: pre-denaturation at 95°C for 5min, 95°C for 30s, annealing to 58°C and holding for 30s, followed by extension at 72°C for 80s for 35 cycles, and finally extension at 72°C for 10 min and storage at 4°C.

Endophytes were amplified by PCR using the universal primers 27F (5'-AGAGTTTGATCCTGGCTCAG-3') and 1492R (5'-TACGGCTACCTTGTTACGACTT-3') of bacteria as primers, and the PCR reaction system was 50μL: 2μL of DNA template, 1μL each of 27F and 1492R, 2×MasterMix 25μL, and add ddH_2O to 50μL. PCR reaction conditions: pre-denaturation at 95°C for 5min, 95°C for 30s, annealing to 55°C for 30s, followed by extension at 72°C for 1min for 35cycles, and finally extension at 72°C for 10min, and storage at 4°C.

After the PCR amplification reaction, the products were detected by the ratio is 1% agarose gel electrophoresis. The amplification products were sequenced by Sangon Biotech.The sequencing results were logged into the official website of NCBI for BLAST comparison analysis, and MEGA11 software was used to create a phylogenetic tree to identify the endophytes.

Determination of bacteriostatic activity of endophytic fungi and endophytic bacterial metabolites

Determination of bacteriostatic activity of endophytic fungi

The pure indicator strains (Staphylococcus aureus, Bacillus subtilis, Escherichia coli) were picked and inoculated onto LB medium by plate streaking method for overnight cultivation; Pick single colonies on LB medium again for plate streaking overnight culture; pick single colonies to 0.85% saline to prepare the bacterial solution, adjust the concentration of the bacterial solution and measured by UV-visible spectrophotometer (UV-5500, Shanghai, China), until OD_{600} = 0.2 ,and the bacterial solution was dipped into cotton swabs to evenly spread onto NA medium to make the bacterial plate containing the indicator strains; a perforator with a diameter of 0.7cm was used to prepare bacterial cakes on the endophytic fungal colonies cultivated for 10 d. The bacterial cakes were placed on the bacterial plate containing the indicator strains in 3 groups of replicates per endophytic fungal strain with blanks. The fungus cake was prepared on the endophytic fungal colonies with a 0.7 cm diameter punch, and placed on the fungus plate containing the indicator species, and 3 cakes were inoculated equidistant from each plate; 3 groups of replicates were made for each endophytic fungus, and the no antibiotics blank drug-sensitive paper(Shandong Best Microbial Technology Co., Ltd.) was used as the blank control, and the culture was incubated at a constant temperature of 28 ℃, and the bacterial inhibitory activity was observed after 3 d. The results were summarized as follows,the size of the ring of inhibition was recorded and the average value was calculated.

Determination of bacteriostatic activity of metabolites of endophytic fungi

The endophytic fungal strain was transferred to a 100 mL conical flask containing 50 mL of PDB liquid medium; the fermentation culture was oscillated at 28 °C and 150 r/min for 7 d. The supernatant was centrifuged at 4 °C and 12000 r/min for 10 min and

then filtered through a 0.22 μm sterile filter membrane, and the fermentation broth was obtained as the metabolites of the endophytic fungi. The blank drug-sensitive paper was placed in the bacterial plate, and each plate was inoculated with 3 pieces of filter paper at equal distance, and 15μL of fermentation broth (endophytic fungal metabolites) was added to each piece of filter paper dropwise by pipette gun; 3 groups of replicates were set up for each endophytic fungal metabolites, and the blank drug-sensitive paper was used as the blank control, and the culture was incubated at a constant temperature of 28 °C, and whether there was any circle of inhibition was observed and the size of the circle of inhibition was measured after 3 d, and the mean value was taken for calculation.

Determination of bacteriostatic activity of endophytic bacterial metabolites

Pick a small amount of pure endophytes, inoculate them into fresh LB medium by plate streaking method, activate and cultivate them at 30℃ for 12~16 h. Pick the activated single colony and transfer it to LB liquid medium, incubate it at 30℃and 230r/min overnight; take 2 mL of the seed solution into 50 mL of the newly formulated LB liquid medium, incubate it at 30℃and 180 r/min for 7 d, and then centrifuge the supernatant after centrifugation for 5 min at 8000 r/min, and filter the fermentation broth obtained by filtration with 0.22μm sterile filter membrane to remove bacteria. After centrifugation, the supernatant was filtered with a 0.22μm sterile membrane to remove bacteria, and the fermentation broth obtained from the filtration was endophytic bacterial metabolites. Determination of bacteriostatic activity of endophytic bacterial metabolites was the same as 1.9.2.

Results and analysis

Results of an orthogonal test on seed disinfection of Panax japonicus

The results of the effects of different disinfection time and disinfectant concentration on the disinfection effect of *Panax japonicus* seeds showed that 75%

alcohol disinfection for 30 s and 1% $HgCl_2$ disinfection for 9 min resulted in the lowest contamination rate of 3.33% (Table 1), and the analysis of extreme variance (ANOVA) indicated that the three factors had the greatest effect on the disinfection effect of *Panax japonicus* seeds as $HgCl_2$, followed by disinfection time and finally 75% alcohol; ANOVA pointed out that the differences between the set ANOVA indicated that the differences among 75% alcohol, $HgCl_2$ and disinfection time on the contamination rate of *Panax japonicus* seeds were not significant at the statistical level within the set gradient range (Table 2). However, it was obtained that $HgCl_2$ (%) > time (min) > 75% alcohol affected seed disinfection in the order of priority (Table 3).

Table 1 Results of the orthogonal test of seed disinfection of *Panax japonicus.japonicus*.

deal with	factor			Results statistics		
	75% alcohol (s)	$HgCl_2$ (%)	Time (min)	Number of inoculations	contami nation	Contamination rate (%)
1	30	0.1	3	30	10	33.33
2	30	0.5	6	30	2	6.67
3	30	1.0	9	30	1	3.33
4	60	0.1	6	30	18	60.00
5	60	0.5	9	30	4	13.33
6	60	1.0	3	30	6	20.00
7	90	0.1	9	30	7	23.33
8	90	0.5	3	30	14	46.67
9	90	1.0	6	30	4	13.33

Table 2 Analysis of variance (ANOVA) of the factors affecting seed disinfection of *Panax japonicus*.

	Type III sum of squares	Degree of freedom	Mean square	F	Signific ance
alcohol	466.667	2	233.333	0.677	0.596
$HgCl_2$	1088.844	2	544.422	1.58	0.388
time	622.444	2	311.222	0.903	0.525
inaccuracies	688.978	2	344.489		

Table 3Extreme variance analysis of factors affecting seed disinfection of *Panax japonicus*.

Item	75% alcohol (s)	$HgCl_2$ (%)	Time (min)
**K1**	43.33	116.67	100.00
**K2**	93.33	66.67	80.00
**K3**	83.33	36.67	40.00
**k1**	14.44	38.89	33.33
**k2**	31.11	22.22	26.67
**k3**	27.78	12.22	13.33
**R**	16.67	26.67	20.00
Order of priority	$HgCl_2$ (%) > time (min) > 75% alcohol		

Isolation and purification of endophytic bacteria from the seeds of *Panax japonicus*

Seven endophytic fungal colonies, named *Pj*Z1, *Pj*Z2, *Pj*Z3, *Pj*Z4, *Pj*Z5, *Pj*Z6, *Pj*Z7, and three endophytic bacterial colonies, named *Pj*X1, *Pj*X2, and *Pj*X3, were isolated from the seeds of *Panax japonicus.*The morphology of all three endophytic bacteria was round, with neat edges and slightly raised centers, the colonies were creamy white, and the results of Gram staining were negative(Table4), the morphology of the fungi is described in Table5.

Fig.1 Characterization of colonies of the endophytic fungi *Pj*Z1-*Pj*Z7 in the seeds of *Panax japonicus*

Fig.2 Characterization of colonies and Gram staining results of endophytic bacteria *Pj*X1, *Pj*X2, and *Pj*X3 from seeds of *Panax japonicus*

Table 4 Morphology and Gram staining of endophytic bacteria in the seeds of

Panax japonicus

Endophytic bacteria	Colony characteristics	Gram stain
*Pj*X1	Rounded, with neat margins and a slightly raised center, the colonies are creamy white	negatives
*Pj*X2	Rounded, with neat margins and a slightly raised center, the colonies are creamy white	negatives
*Pj*X3	Rounded, with neat margins and a slightly raised center, the colonies are creamy white	negatives

Table 5 Characteristics of endophytic fungal colonies in the seeds of *Panax japonicus.*

Endophytic fungi	Appearance	Microscopic form
*Pj*Z1	Mycelium abundant, aerial hyphae well-developed; colonies raised, tomentose, white on the front, orange on the back, with goose-yellow margins.	Mycelium septate, branched; spores green,rounded;broom-like spore-producing structures.
*Pj*Z2	Mycelium fewer, aerial hyphae well developed; colonies raised, tomentose, adaxial margin yellow, middle pink, abaxial light yellow-brown, margin goose-yellow.	Mycelium septate, branched; spores green,rounded;broom-like spore-producing structures.
*Pj*Z3	Mycelium abundant, aerial hyphae well-developed; colonies raised, tomentose, white adaxially, light yellow abaxially.	Mycelium septate, branched; spores green,rounded;broom-like spore-producing structures.
*Pj*Z4	Mycelium abundant, aerial hyphae well-developed; colonies raised, tomentose, gray-yellow adaxially, brown abaxially, with goose-yellow margins.	Mycelium septate, branched; spores colorless,rounded;broom-like spore-producing structures.
*Pj*Z5	Mycelium abundant, aerial hyphae well developed; colonies raised, cottony, white on the front, goose-yellow on the back.	Mycelium septate, branched; spores green,rounded;broom-like spore-producing structures.
*Pj*Z6	Mycelium fewer, aerial hyphae well developed; colonies raised, downy, pink on the front, light orange on the back.	Mycelium septate, branched; spores green,rounded;broom-like spore-producing structures.
*Pj*Z7	Mycelium abundant, aerial hyphae well-developed; colonies raised, tomentose, grayish-white adaxially, brown in the middle of the abaxial surface, grayish-brown at the edges.	Mycelium septate, branched; spores colorless,rounded;broom-like spore-producing structures.

Molecular biological identification and homology analysis of endophytic bacteria

ITS sequence analysis indicated that the sequence lengths of seven endophytic

fungi were *Pj*Z1 511bp, *Pj*Z2 459bp,*Pj*Z3 502bp,*Pj*Z4 489bp,*Pj*Z5 607bp,*Pj*Z6 519bp, and *Pj*Z7 512bp, and the size of the target bands amplified by PCR using the universal primers ITS1 and ITS4 (Fig.3) was in agreement with the sequencing results. The 16SrDNA sequence analysis indicated that the sequence lengths of the three endophytic bacteria were *Pj*X1 1413bp, *Pj*X2 1400bp, and *Pj*X3 1416bp, respectively, and the size of the target bands amplified by PCR using the universal primers 27F and 1492R (Fig.4) was consistent with the sequencing results.BLAST search was performed at NCBI and a Utilization of the software MEGA11 phylogenetic tree was constructed (Fig.5), which revealed that the sequence similarity between endophytic fungus *Pj*Z1 and accession number JX827458.1 was 100% and identified as *Fusarium tricinctum*;*Pj*Z2 and accession number MT601889.1 had 100% sequence similarity and identified as *Fusarium reticulatum*; *Pj*Z3 showed 100% sequence similarity to accession number JX401981.1 and was identified as *Fusarium tricinctum*; *Pj*Z4 showed 100% sequence similarity to accession number OR671459.1 and was identified as *Fusarium tricinctum*;*Pj*Z5 showed 96.67% sequence similarity with accession number OR543773.1 and was identified as *Fusarium tricinctum*;*Pj*Z6 showed 100% sequence similarity with accession number LT746261.1 and was identified as *Fusarium sarcochroum*; *Pj*Z7 showed 100% sequence similarity with accession number LT746261.1 with 100% sequence similarity, identified as Streptomyces *alternaria alternata*. endophytic bacteria *Pj*X1 with accession number JQ864378.1 with 100% sequence similarity, identified as *Enterobacteriaceae bacterium*,*Pj*X2 with accession number PQ097710.1 with 99.93% sequence similarity and identified as *Pseudomonas aeruginosa*, *Pj*X3 with 99.79% sequence similarity to accession number JX067703.1 and identified as *Enterobacteriaceae* bacterium.

Fig3 PCR identification of endophytic fungi in the seeds of *Panax japonicus*.

Marker:DNA Marker DL2000; 1-3 for *Pj*Z1, 4-6 for *Pj*Z2, 7-9 for *Pj*Z3, 10-12 for *Pj*Z4, 13-15 for *Pj*Z5, 16-18 for *Pj*Z6, 19-21 for *Pj*Z7

Fig4 PCR identification of endophytic bacteria in the seeds of *Panax japonicus*.

Marker:DNA Marker DL2000; 1-3 for *Pj*X1, 4-6 for *Pj*X2, 7-9 for *Pj*X3

Fig5 Phylogenetic tree of endophytes

a. Phylogenetic tree constructed from *Pj*Z1-*Pj*Z7 rDNA-ITS sequences of the endophytic fungi from the seeds of *Panax japonicus*,

b. Phylogenetic tree constructed from the rDNA sequences of endophytic bacteria *Pj*X1-*Pj*X3 in the seeds of *Panax japonicus*

Determination of bacteriostatic activity of endophytic fungal bacteria

Determination of bacteriostatic activity were carried out on seven strains of endophytic fungi isolated with Escherichia coli, Staphylococcus aureus and Bacillus subtilis, and the results showed that (Table 6), strain *Pj*Z4 had inhibitory activity against Escherichia coli, but the inhibitory activity was weak, with an average inhibitory circle diameter of 10.48 mm, and the remaining six strains of the fungi did not have any inhibitory activity against E. coli; the seven strains of the endophytic fungi strains did not have any inhibitory activity against Staphylococcus aureus; Strain *Pj*Z3, *Pj*Z4, *Pj*Z5, *Pj*Z7 had bacteriostatic activity against B.subtilis, strain *Pj*Z3 had the strongest bacteriostatic activity against B. subtilis, with an average inhibitory circle diameter of 23.53mm, strain *Pj*Z4 had the weakest inhibitory activity against B. subtilis, with an average inhibitory circle diameter of 13.69mm, and the average inhibitory circle diameters of strains *Pj*Z5 and *Pj*Z7 were respectively 16.80mm, 17.97mm, strains *Pj*Z1, *Pj*Z2 and *Pj*Z6 had no inhibitory activity against B. subtilis, and no inhibitory circle was produced in the control blank drug-sensitive paper sheet (Figure 6).

Fig6 Inhibitory activity of endophytic fungi from the seeds of *Panax japonicus* against three pathogenic bacteria

A、B and C are blank controls for the three pathogens; D, *Pj*3/Bacillus subtilis; E, *Pj*4/E. coli; F, *Pj*4/Bacillus subtilis; G, *Pj*5/Bacillus subtilis; and H, *Pj*7/Bacillus subtilis.

Table 6 Inhibitory activity of endophytic fungi from the seeds of *Panax japonicus* against three pathogenic bacteria

Strain number	Escherichia coli (E. coli)	Staphylococcus aureus	Bacillus subtilis
	Diameter of inhibition circle/ mm		
*Pj*Z1	-	-	-
*Pj*Z2	-	-	-
*Pj*Z3	-	-	23.53
*Pj*Z4	10.48	-	13.69
*Pj*Z5	-	-	16.80
*Pj*Z6	-	-	-
*Pj*Z7	-	-	17.97

Bacteriostatic activity of endophyte metabolites

Antagonistic assay of the metabolites of 7 strains of endophytic fungi and 3 strains of bacteria isolated against Escherichia coli, Staphylococcus aureus and Bacillus subtilis showed that (Table7), the metabolites of the 7 strains of endophytic fungi had no inhibitory activity against Escherichia coli, Staphylococcus aureus and Bacillus subtilis, and there was no inhibitory circle on the control blank drug-sensitive paper sheet as well.

Antagonistic assay of the metabolites of the three isolated endophytic bacteria with E. coli, S. aureus and B. subtilis revealed that the metabolites of the three endophytic bacteria had no bacteriostatic activity against E. coli, S. aureus and B. subtilis.(Table8)

Table 7 Inhibitory activity of endophytic fungal metabolites from the seeds of *Panax japonicus*

Strain number	Escherichia coli (E. coli)	Staphylococcus aureus	Bacillus subtilis
*Pj*Z1	—	—	—
*Pj*Z2	—	—	—
*Pj*Z3	—	—	—
*Pj*Z4	—	—	—
*Pj*Z5	—	—	—
*Pj*Z6	—	—	—
*Pj*Z7	—	—	—

Table 8 Inhibitory activity of endophytic fungal metabolites from the seeds of *Panax japonicus*

Strain number	Escherichia coli (E. coli)	Staphylococcus aureus	Bacillus subtilis

*Pj*Z1	—	—	—
*Pj*Z2	—	—	—
*Pj*Z3	—	—	—
*Pj*Z4	—	—	—
*Pj*Z5	—	—	—
*Pj*Z6	—	—	—
*Pj*Z7	—	—	—

Discussion

In recent years, the germplasm resources of *Panax japonicus* have been endangered, and the conservation of the germplasm resources of *Panax japonicus* has become a key issue to be solved. In the process of artificial breeding, we found that the seeds of *Panax japonicus* were difficult to germinate and the seeds of *Panax japonicus* were difficult to germinate., studies have shown that different plant seed endophytes can potentially increase the productivity of host plants[12]. However, the isolation and identification of endophytic fungi from the seeds of *Panax japonicus* have not been reported.In this study, seven strains of endophytic fungi were isolated from the seeds of *Panax japonicus*, among which six strains belonged to *Fusarium,*and one strain belonged to *Alternaria alternata* The two genera of fungi had almost no inhibitory effect on Escherichia coli and Staphylococcus aureus, and had inhibitory activity on bacillus cereus but the inhibitory effect was not obvious, and the metabolites of the two genera had no inhibitory activity on the indicator organisms. The metabolites of the two genera of fungi had no bacteriostatic activity on the indicator bacteria, indicating that the two genera of fungi were not the dominant species of endophytic fungi in the seeds of *Panax japonicus*. And many studies pointed out that *Fusarium*. and *Alternaria alternata* are pathogenic fungi that cause plant diseases, such as Yang isolation and identification of pathogenic fungi on naturally-occurring *Passiflora edulis*, and found that the pathogenic fungi that cause post-harvest *Passiflora edulis* diseases belong to *Fusarium* spp. and *Streptomyces* .[13] Zhong determined the pathogenicity of different strains of *Fusarium* species on *Nicotiana tabacum*, and showed that the pathogenic

fungi of *Fusarium* root rot of *Nicotiana tabacum* were mainly shared by *Fusarium*., and 40% of imipramine EW had an inhibitory effect on this pathogen[14].Chen isolated and identified the pathogenic bacteria from the leaves of the diseased plants of *Pseudocercospora variicola*, and the results showed that the pathogen of *Pseudocercospora variicola* was *Alternaria alternata*.[15].All these studies confirmed that *Fusarium*. and *Alternaria alternata*. are not dominant species in plants.still ,both of them cause different diseases and inhibit the normal growth and development of plants in various degrees.Therefore, it is likely that the difficulty in seed germination of *Panax japonicus* is due to the action of these two endophytic fungi, and further research is needed to find antibiotics to inhibit the growth of this endophytic fungus in order to solve the problem of the difficulty in seed germination of *Panax japonicus*.

In this study, three strains of endophytic bacteria were also isolated from the seeds of *Panax japonicus*, two of which belonged to *Enterobacteriaceae* and one belonged to *Pseudomonas aeruginosa*, and the metabolites of the two genera of bacteria had no inhibitory activity against the indicator bacteria, which may be due to the high concentration of the indicator bacteria or the low activity of the metabolites. Some studies have shown that Enterobacteriaceae genus has a better effect in promoting plant growth, such as Zhao isolated endophytic Enterobacteriaceae from the *Paspalum vaginatum* as a material to explore the germination of dogbane seeds and salt tolerance after ping, the results of the seed germination rate and salt tolerance after ping has been improved.[16] Some scholars isolated endophytic bacteria from the root system of cucumber, and found that Enterobacteriaceae and Pseudomonas spp. played an important role in the control of cucumber wilt disease[17].Ma initially identified eight strains of bacteria isolated from plant roots and investigated the growth-promoting effects of endophytic bacteria on ryegrass, which showed that all eight strains of bacteria belonged to *Pseudomonas aeruginosa*. and could effectively promote the growth and development of *Lolium perenne*[18].The results showed that all eight strains of bacteria belonged to Pseudomonas spp. From the results of many studies, it can be inferred that *Enterobacteriaceae* and *Pseudomonas aeruginosa*. are the dominant strains in promoting plant growth and development, therefore, further investigation can

be carried out in the future seed germination and disease control of *Panax japonicus.*

Conclusion

The orthogonal experiment screened out the optimal disinfection conditions of *Panax japonicus* seeds as 75% alcohol disinfection for 30s and 1% $HgCl_2$ disinfection for 9min, which had the lowest contamination rate of 3.33%. Seven strains of endophytic fungi *Pj*Z1, *Pj*Z2, *Pj*Z3, *Pj*Z4, *Pj*Z5, *Pj*Z6, *Pj*Z7 and three strains of *endophytic bacteria Pj*X1, *Pj*X2, *Pj*X3 were isolated and screened from the seeds of *Panax japonicus*, and the strains of *Pj*Z1, *Pj*Z3, *Pj*Z4, *Pj*Z5 were identified as the fungi of the *Fusarium* genus, and the strains of *Pj*Z2 and *Pj*Z4 were identified as the fungi of the *Fusarium* genus. *Fusarium reticulatum*, *Pj*Z6 is *Fusarium sarcochroum*, *Pj*Z7 is Alternaria alternata; *Pj*X1 and *Pj*X3 are *Enterobacteriaceae bacterium*, and *Pj*X2 is *Pseudomonas aeruginosa*. The results of inhibition experiments showed that only *Pj*Z4 had inhibitory activity against E. coli, *Pj*Z3, *Pj*Z4, *Pj*Z5, *Pj*Z7 had inhibitory activity against Bacillus subtilis, and none of the 7 endophytic fungal strains had inhibitory activity against Staphylococcus aureus, and the metabolites of the 10 endophytic strains of endophytic fungi were antagonized with E. coli, Staphylococcus aureus, and Bacillus subtilis. The experiment showed that the metabolites of 10 strains of endophytic bacteria had no bacteriostatic activity against Escherichia coli, Staphylococcus aureus and Bacillus subtilis. After preliminary judgment, the isolated *Fusarium spp.* and *Alternaria alternata*. are not the dominant species in the growth and development process of *Panax japonicus*, and it can be inferred that the difficulty in seed germination of bamboo ginseng may be caused by the growth of these two genera of fungi inhibiting the

synthesis of growth hormone by the seeds during the seed germination process. The isolated *Enterobacteriaceae bacterium* and *Pseudomonas spp*. are the dominant species in promoting plant growth and development, so these two genera of bacteria can be utilized in the cultivation process of *Panax japonicus* to promote its growth and development.The endophytes isolated in this study provide a theoretical basis for the screening of endophyte antagonist strains of *Panax japonicus* and for seed germination and disease control of *Panax japonicus*.

Acknowledgements

We would like to thank Professor Lai Zhang for his careful guidance of this study ,School of Life Science, Guizhou Normal University and the Guizhou Provincial Innovation Center for Mountainous and Efficient Agriculture for its strong support.

We thank the editors and reviewers for their hard work on our papers.

Reference

[1] ZHOU Jing, FU Xian Yun, CAI San Jin, YUAN Ding, MEI Zhigang, FENG Zhitao. [2]Research progress on chemical constituents and pharmacological effects of *Panax japonicus* and predictive analysis on quality markers [J]. Chinese Herbal Medicine,2021,52(09):2819-2830

[2] SHEN Peiyao, ZHANG Ye, LI Yuqin, YAN Jianye, WANG Yuanqing. [3]Research progress on biological activity and quality control of *Panacis Japonici Rhizoma*[J]. Food and Machinery,2021,37(11):211-220.

[3] GUO Zhe, FENG Zhitao, ZHANG Haoran, YAN Lan, LIANG Mingge, MEI Zhigang, CAI Sanjin. Progress of research on *Panax japonicus* and its preparation for the treatment of rheumatoid arthritis[J]. Chinese Materia Medica,2019,42(04):941-944.

[4] LI Chunyan, ZHANG Jie, LI Jinping, et al. [5]Research on Chemical Constituents and Biological Activities of Rhizoma of *Panax japonicus*[J]. Introduction to Traditional Chinese Medicine,2012,18(04):68-71.DOI:10.13862/j.cnki.cn43-1446/r.2012.04.050.

[5] YANG Fajian,DING Guoyu,WANG Hongyun,et al. Technical points of seed multiplication of the rare plant Bamboo Ginseng[J]. Southern Agriculture,2020,14(29):19-20. DOI:10.19415/j.cnki.1673-890x.2020.29.010.

[6] CHENG Longyuan, ZHANG Guohui, SUN Yan, et al. Progress of medicinal plant-endophyte-inter-root microbial interactions[J]. Chinese Herbal Medicine,2024,55(15):5264-5273.

[7] Liang E.Endophytic Bacterial Diversity and Cloning＆BioinformaticsAnalysis of Squalene epoxidase(SE)Gene in *Panax japonicusC.A.Meyer*[D]. Guizhou Normal University,2020.DOI:10.27048/d.cnki.ggzsu.2020.000068.

[8] ZHU Lili,ZHANG Yemeng,LI Wancai,et al. Comprehensive evaluation of the effects of different disinfectants on surface disinfection and germination of quinoa seeds[J]. Journal of Biology,2023,40(4):83-89. DOI:10.3969/j.issn.2

095-1736.2023.04.083.

[9] ZHANG Yubai, WEN Xiangshui, WANG Yumeng, et al. Isolation and characterization of endophytic fungi from the seeds of Fructus schisandrae chinensis and their fungicidal properties[J]. Chinese Materia Medica,2020,43(5): 1087-1091. DOI:10.13863/j.issn1001-4454.2020.05.008.

[10] Wei, Jingchao. Handbook of fungal identification [M]. Shanghai: Shanghai Science and Technology Press, 1979.09.

[11] Dong Xiuzhu, Cai Miaoying, et al. eds. Handbook of Systematic Identification of Common Bacteria [M]. Beijing: Science Press, 2001.02.

[12] Liang Danni. Mechanisms of Seed Endophytic BacteriaRegulating Nitrogen Transformation[D]. Lanzhou University,2023.DOI:10.27204/d.cnki.glzhu.2023.004000.

[13] YANG Dingzhao,SHUAI Liang,TANG Suizhi,et al. Isolation and identification of passion fruit derived pathogenic bacteria and preliminary study on inhibitory activity of bactericidal agents[J/OL]. Food and Fermentation Industry,1-12[2024-08-19].https://doi.org/10.13995/j.cnki.11-1802/ts.040144.

[14] ZHONG Jie, QIU Zelan, LIU Shida, et al. Pathogen Identification of Tobacco Root Rot Disease in Hunan Province and Screening of Control Fungicides[J]. China Tobacco Science,2024,45(03):51-59+67.DOI:10.13496/j.issn.1007-5119.2024.03.008.

[15] CHEN Cun,LIU Yan,ZHAO Yuyang,et al. Isolation and characterization of the pathogenic bacteria of Verticillium verticillioides in Paeonia lactiflora[J/OL]. Chinese Materia Medica,2024,(07):1630-1633[2024-08-19].https://doi.org/10.13863/j.issn1001-4454.2024.07.005.

[16] ZHAO Xintong,CHEN Xiaodong,LI Ziji,et al. An evaluation of the effects of the plant endophyte Enterobacter on the salt tolerance of bermudagrass [J]. Journal of Grass Industry,2021,30(09):127-136.

[17] Yu-Lu Zhang 1,Xiao-Jing Guo 1,Xin Huang 1 2,Rong-Jun Guo 1,Xiao-Hong Lu 1,Shi-Dong Li 1,Hao Zhang 2.The Co-Association of Enterobacteriaceae and Pseudomonas with Specific Resistant Cucumber against Fusarium W

ilt Disease[J].Biology.2023,Vol.12(No.2):143.
[18]MA Yachun,YAO Tuo,LEI Yang,et al. Screening of pseudomonas inorganic phosphate and its effect on ryegrass growth[J]. Grassland and Turf,2024,44 (03):101-107.DOI:10.13817/j.cnki.cyycp.2024.03.013.

Supporting Information

S1_raw_images

Click here to access/download;Figure;Fig1.tif.docx

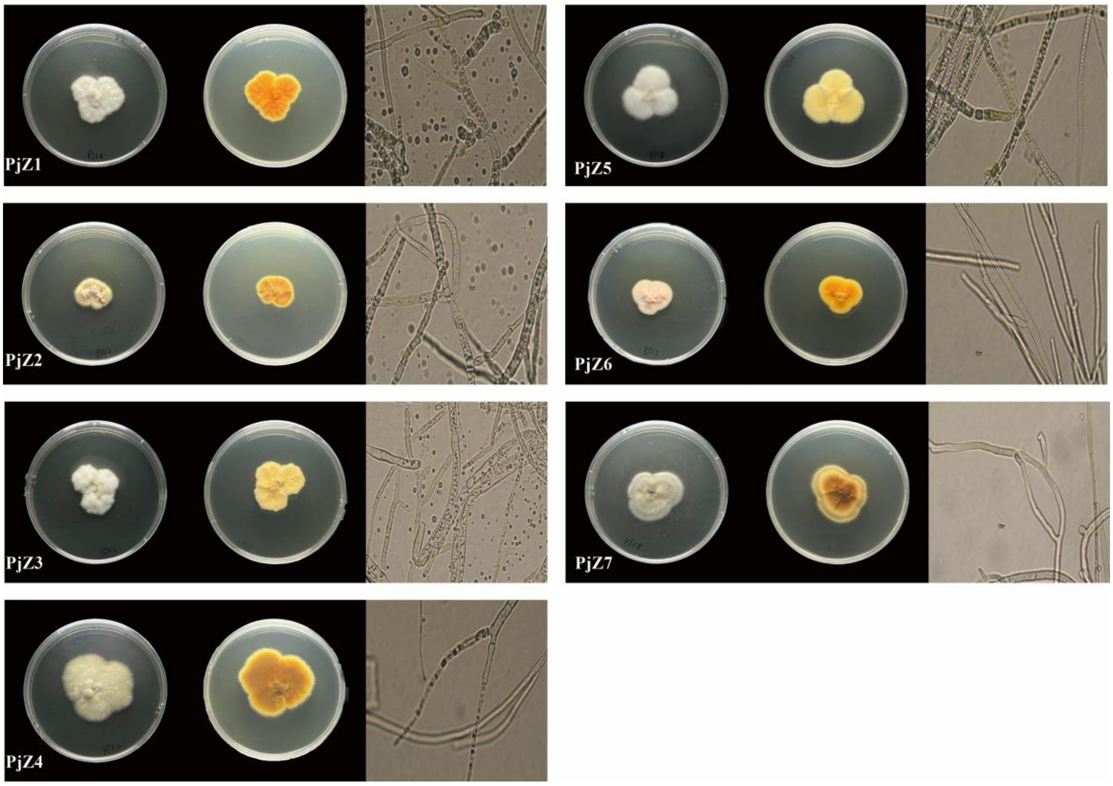

Click here to access/download;Figure;Fig2.tif.docx

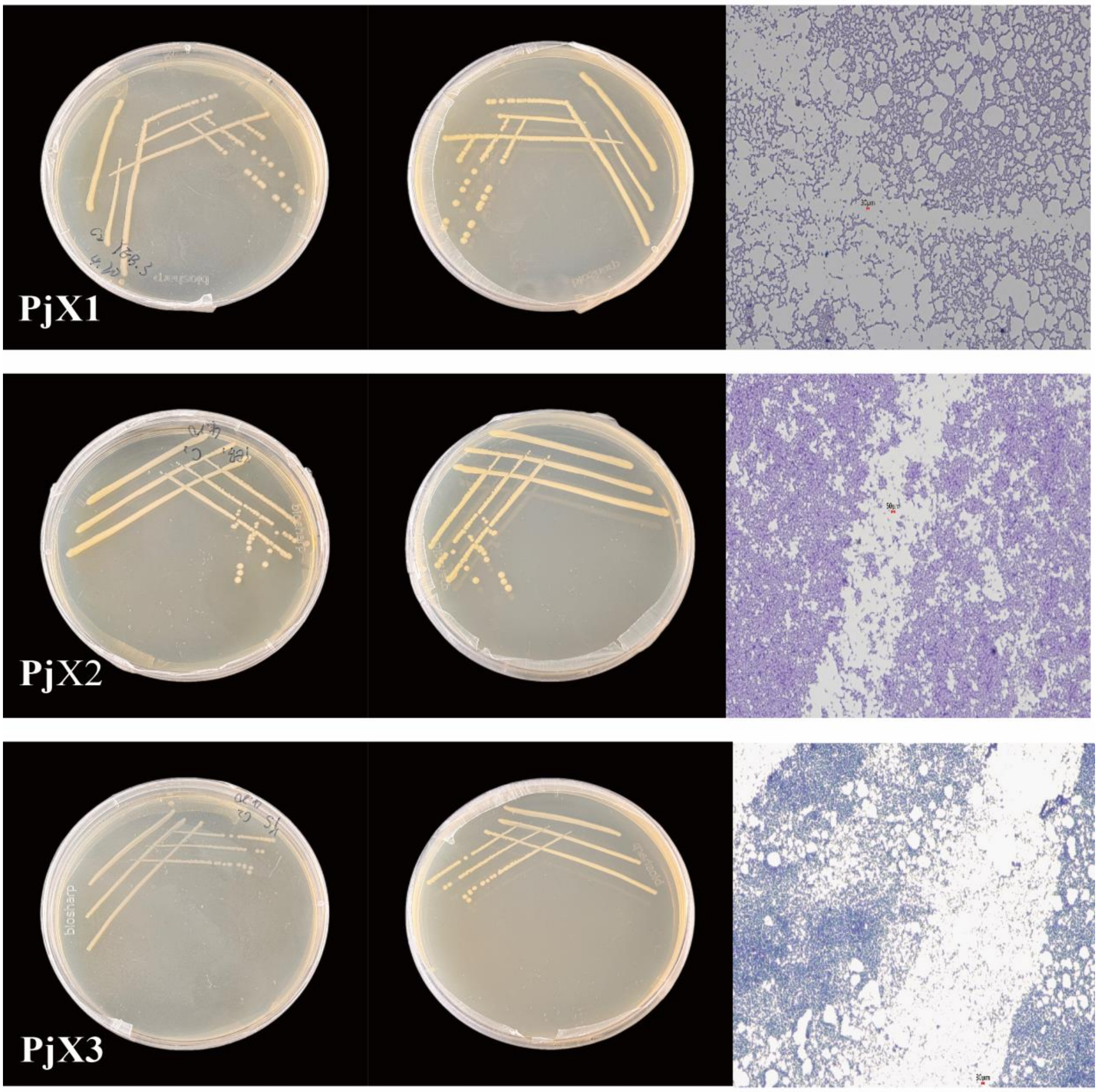

Click here to access/download;Figure;Fig3.tif.docx

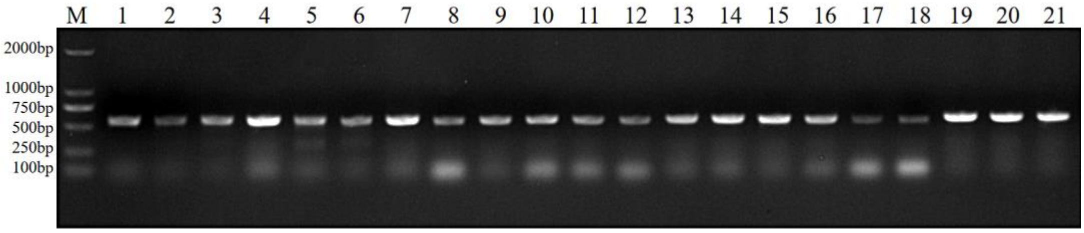

Click here to access/download;Figure;Fig4.tif.docx

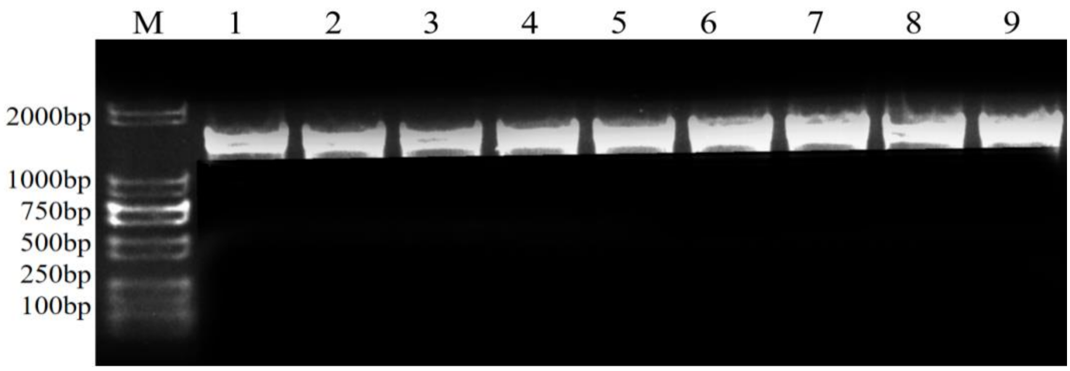

Click here to access/download;Figure;Fig5.tif.docx

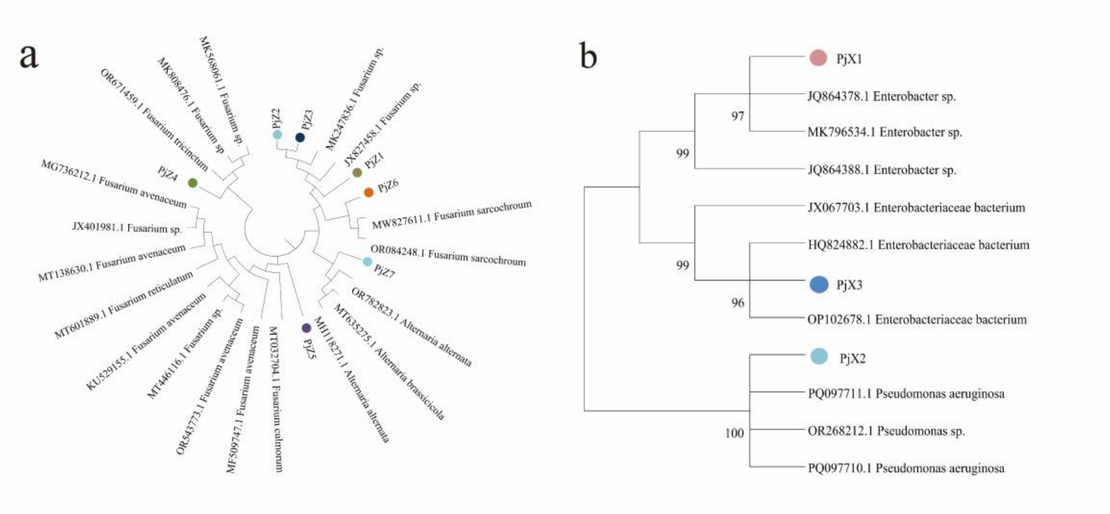

Click here to access/download;Figure;Fig6.tif.docx

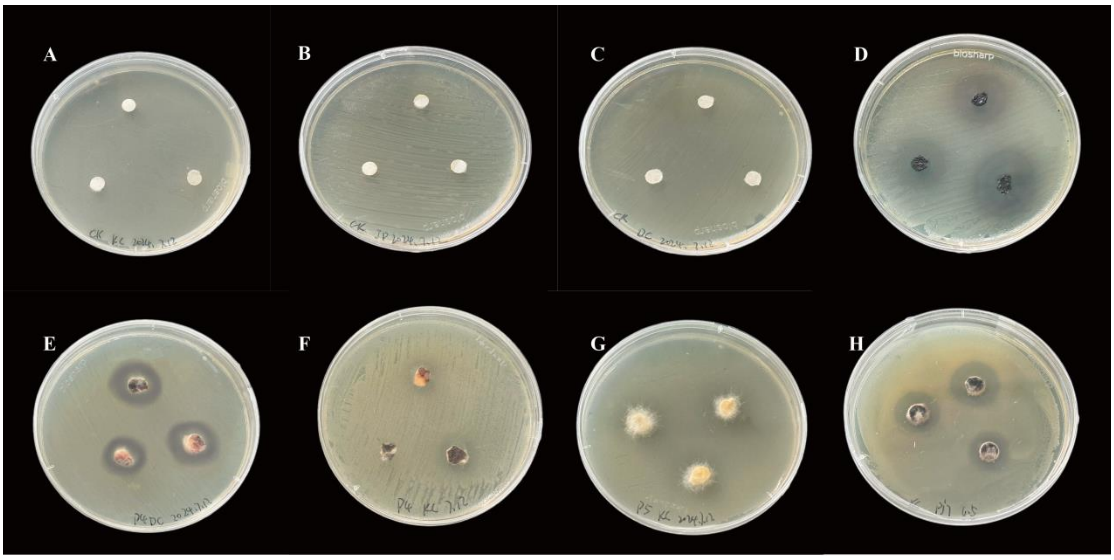

Click here to access/download
Supporting Information
S1_raw_images.pdf

Isolation, identification and antibacterial activity of endophyte from the seeds of *Panax japonicus*

Rui Jin [1,2*] ,Tingting Tang [1,2*] , Xilun Huang [1,2] , Juan Huang [2] , E Liang [2] , Lai Zhang [2*]

[1]School of Life Science, Guizhou Normal University,Guizhou,Guiyang,China;

[2]Anshun university/Innovation Center for Efficient Agricultural of Guizhou Mountain Characteristics,Guizhou,Anshun,China

*Corresponding author

E-mail:975575681@qq.com.(LZ)

Author contribution statement:

Rui Jin(first author): Determine experimental steps, conduct experiments, software analysis, writing - manuscript;

Tingting Tang(co-first author) : Data collation, writing - original manuscript;

Xilun Huang: Image collation and analysis, sequence analysis;

Juan Huang: Software application and result verification

E Liang: Writing - reviewing and editing;

Lai Zhang(corresponding author): Conceptualization, funding acquisition, resources,

supervision, article revision and review.

Abstract

The purpose of this study is to isolate and identify endophytic bacteria from the seeds of *Panax japonicus*, finding a dominant strain that favors seed germination in *Panax japonicus* and to provide theoretical reference for further research on the effect of endophytic bacteria on seed germination. **Methods:** The endophytic bacteria were isolated from the seeds by plate separation method. The isolated endophytes were identified by morphology, ITS and 16S rDNA sequence analysis.The antibacterial activity of the isolated endophytes and their metabolites was studied by disk method. **Results:** The seed was disinfected with 75% alcohol for 30s and 1% $HgCl_2$ for 9min, and the contamination rate was the lowest (3.33%). Seven endophytic fungi strains were isolated from the seeds of *Panax japonicus*, named *Pj*Z1, *Pj*Z2, *Pj*Z3, *Pj*Z4, *Pj*Z5, *Pj*Z6, and *Pj*Z7, respectively. Four strains belonged to the genus *Fusarium tricinctum,* one to *Fusarium reticulatum,* one to *Fusarium sarcochroum,* and one to *Alternaria alternata*; Three strains of endophytic bacteria were isolated and named *Pj*X1, *Pj*X2 and *Pj*X3, respectively. After morphological observation and 16S rDNA gene sequencing, two strains belong to the genus *Enterobacteriaceae bacterium* and one belongs to the genus *Pseudomonas sp*. Among the endophytic fungal strains isolated, only *Pj*Z4 showed significant bacteriostatic activity against *Escherichia coli*, *Pj*Z3, *Pj*Z4, *Pj*Z5 and *Pj*Z7 had antibacterial activity against Bacillus subtilis, and none of the 7 strains had antibacterial activity against Staphylococcus aureus. It was found that the metabolites of 7 endophytic fungi and 3 endophytic bacteria had no antibacterial activity. **Conclusion:** In this study, we investigated the method of bamboo ginseng seed disinfection, and the results showed that 75% alcohol disinfection for 30s and 1% $HgCl_2$ disinfection for 9min resulted in the lowest contamination rate of 3.33%, in which the greatest influence on the seed disinfection effect was the concentration of $HgCl_2$. Meanwhile, 11 endophyte species of *Panax japonicus* seeds were isolated and identified, *Fusarium spp.* and *Alternaria alternata.* were not the dominant species in the growth and development process of *Panax japonicus* while *Enterobacteriaceae* and *Pseudomonas sp.* were the dominant species in the promotion of plant growth and

development, and provides a theoretical reference for further research on the biological functions and active substances of endophytic fungi in the seeds of *Panax japonicus*.

Keywords:*Panax japonicus*; Seeds; Endophyte; Isolation and identification; Endophytic antibacterial activity

Itroduction

Panax japonicus is the dried rhizome of *Panax japonicus* C.A. Mey..The chemical composition mainly includes saponins, sugars, amino acids, volatile oils, flavonoids, nucleosides and inorganic salts, etc.[1].Pharmacological studies have shown that *Panax japonicus* has a good therapeutic effects in enhancing immunity, anti-fatigue, cardioprotection, anti-tumor, anti-myocardial ischemia, sedation and analgesia, and treatment of rheumatoid arthritis[2-4]. Because of the high efficacy and high price of the rare wild Chinese herbal medicine, it is almost difficult to find wild resources of *Panax japonicus* in the wild, so the artificial propagation of *Panax japonicus* seeds is urgent[5].Therefore, the selection of *Panax japonicus* seeds, leaves and stems for seedling propagation is a better choice of explants. It is noteworthy that the seeds of *Panax japonicus* have the phenomenon of after-ripening, and the contamination rate is high during the germination process of histoculture, which makes the germination difficult. Therefore, we carried out further exploration. It has been shown that endophytes of medicinal plants are important for promoting the growth of medicinal plants, improving the quality of medicinal plants, and eliminating the barriers of continuous cultivation[6].Scholars have isolated endophytic bacteria from roots, stems, leaves, fruits,and rhizomes of *Panax japonicus*[7].However, the isolation of endophytic fungi and bacteria from the seeds of *Panax japonicus* has not yet been reported. Therefore, the isolation of endophytic bacteria from the seeds of *Panax japonicus* and their classification, characterization,and bacteriostatic studies can provide certain theoretical references for the further development of the research on the biological functions and active substances of the endophytic bacteria in the seeds of *Panax japonicus*, as well as for the germination of the seeds of *Panax japonicus.*

Materials and Methods

Plant material

Fresh seeds of *Panax japonicus* were obtained from the experimental site of *Panax japonicus* cultivation in the "Guizhou Innovation Center of Mountain Specialty and Efficient Agriculture" of Anshun College, Anshun City, Guizhou Province, China.

Culture medium

Fungal isolation medium: Potato Dextrose Agar Medium (PDA solid medium) 40.1g, distilled water 1000mL, pH:5.8. Bacterial isolation medium: Beef Extract Peptone Medium (NA solid medium): Peptone 10g, Beef Extract 3g, Sodium Chloride 5g, Agar 20g, distilled water 1000mL, pH:7.2. Experimental Medium for Fungal Inhibition Nutrient broth (NB liquid medium): peptone 10g, Beef Extract 3g, sodium chloride 5g, distilled water 1000mL, pH: 7.2, Beef Extract Peptone Medium (NA solid medium). Fungal fermentation medium: PDB liquid medium, Beef Extract Peptone Medium (NA solid medium), nutrient broth (NB liquid medium). Bacterial fermentation medium: LB solid medium, LB liquid medium, MH medium.

Bacterial strain

Strains Staphylococcus aureus, Escherichia coli, Bacillus subtilis, purchased from Guangdong Huankai Biotechnology Co.

Screening of seed disinfection conditions

With 75% alcohol disinfection time of 30s, 60s, 90s, $HgCl_2$ concentration of 0.1%, 0.5%, 1.0%, $HgCl_2$ disinfection time of 6min, 12min, 18min as the influencing factors, a 3-factor, 3-level orthogonal test was designed according to the orthogonal table of L_9 (3^3), with a total of 9 treatments[8].The results of the experiment were analyzed using the software SPSS.

Disinfection

Prior to the isolation of endophytes from the seeds of *Panax japonicus*, the seeds

of *Panax japonicus* were sterilized under different conditions and the contamination rate under different sterilization conditions were analyzed.The seeds were rinsed under running water and then sterilized with the above orthogonal design combinations.After disinfection, the seeds were inoculated on MS medium and the contamination rate was counted.

Isolation and purification of endophytic fungi

Take the fresh seeds of *Panax japonicus* and wash them under running water, then rinse them 1-3 times with sterile water, disinfect them with 75% alcohol for 30s, 1% $HgCl_2$ for 9min, rinse them with sterile water for 2-3 times and then dry them with sterile filter paper of 5 cm^2 in size, peel them and then cut them in half, put them on PDA medium with the cut side facing down[9], and then place them in an incubator at 28℃ for cultivation. After the growth of mycelium, pick the mycelium for repeated isolation and purification for 2-3 times for preservation of the isolated strains.

Isolation and purification of endophytic bacteria

Take fresh seeds of *Panax japonicus* and rinse them under running water, then rinse them 1-3 times with sterile water, the same as 1.6 Seed Disinfection Method, peel the seeds and cut them in half, place them on NA medium with the cut side facing down, and place them in the incubator at 28℃ for cultivation. After the growth of colonies, pick the strains and line the purification, purify 2-3 times to keep the seeds.

Identification of endophytes

Morphological observation

Pick the purified fungal hyphae and make them into clinical slices, observe and record (hyphae color, hyphae morphology, spore morphology, etc.) through an optical microscope, and refer to "Fungal Identification Manual".[10] etc. for preliminary identification of fungi. (Three strains on each medium for each strain and three strains on each medium for observation and identification).The purified bacterial strains were

subjected to continuous observation, and records were made (diameter size, color, morphology, dryness, whether transparent or not, etc. of the colonies). Pick a small amount of bacterial organisms to make clinical slices for Gram staining, and refer to the "Manual of Identification of Common Bacterial Systems" for preliminary analysis and identification[11].Make preliminary analysis and identification.

Molecular biology identification: Endophytic fungal DNA was extracted according to the instructions of the fungal DNA extraction kit (Solarbio), and endophytic bacterial DNA was extracted according to the instructions of the bacterial DNA extraction kit. Subsequently, PCR amplification was performed using the DNA of each endophytic bacterium as a template, respectively.

Endophytic fungi were amplified by PCR using the universal primers of fungi, ITS1 (5'-TCCGTAGGTGAACCTGCGG-3') and ITS4 (5'-TCCTCCGCTTATTGATATGC-3') as primers, and the total volume of the PCR reaction system was 50μL, containing:4μL of DNA template, 2μL of each of ITS1 and ITS4, and 2×MasterMix 25μL, and add ddH_2O 17μL. PCR reaction conditions: pre-denaturation at 95°C for 5min, 95°C for 30s, annealing to 58°C and holding for 30s, followed by extension at 72°C for 80s for 35 cycles, and finally extension at 72°C for 10 min and storage at 4°C.

Endophytes were amplified by PCR using the universal primers 27F (5'-AGAGTTTGATCCTGGCTCAG-3') and 1492R (5'-TACGGCTACCTTGTTACGACTT-3') of bacteria as primers, and the PCR reaction system was 50μL: 2μL of DNA template, 1μL each of 27F and 1492R, 2×MasterMix 25μL, and add ddH_2O to 50μL. PCR reaction conditions: pre-denaturation at 95°C for 5min, 95°C for 30s, annealing to 55°C for 30s, followed by extension at 72°C for 1min for 35cycles, and finally extension at 72°C for 10min, and storage at 4°C.

After the PCR amplification reaction, the products were detected by the ratio is 1% agarose gel electrophoresis. The amplification products were sequenced by Sangon Biotech.The sequencing results were logged into the official website of NCBI for BLAST comparison analysis, and MEGA11 software was used to create a phylogenetic tree to identify the endophytes.

Determination of bacteriostatic activity of endophytic fungi and endophytic bacterial metabolites

Determination of bacteriostatic activity of endophytic fungi

The pure indicator strains (Staphylococcus aureus, Bacillus subtilis, Escherichia coli) were picked and inoculated onto LB medium by plate streaking method for overnight cultivation; Pick single colonies on LB medium again for plate streaking overnight culture; pick single colonies to 0.85% saline to prepare the bacterial solution, adjust the concentration of the bacterial solution and measured by UV-visible spectrophotometer (UV-5500, Shanghai, China), until OD_{600} = 0.2 ,and the bacterial solution was dipped into cotton swabs to evenly spread onto NA medium to make the bacterial plate containing the indicator strains; a perforator with a diameter of 0.7cm was used to prepare bacterial cakes on the endophytic fungal colonies cultivated for 10 d. The bacterial cakes were placed on the bacterial plate containing the indicator strains in 3 groups of replicates per endophytic fungal strain with blanks. The fungus cake was prepared on the endophytic fungal colonies with a 0.7 cm diameter punch, and placed on the fungus plate containing the indicator species, and 3 cakes were inoculated equidistant from each plate; 3 groups of replicates were made for each endophytic fungus, and the no antibiotics blank drug-sensitive paper(Shandong Best Microbial Technology Co., Ltd.) was used as the blank control, and the culture was incubated at a constant temperature of 28 ℃, and the bacterial inhibitory activity was observed after 3 d. The results were summarized as follows,the size of the ring of inhibition was recorded and the average value was calculated.

Determination of bacteriostatic activity of metabolites of endophytic fungi

The endophytic fungal strain was transferred to a 100 mL conical flask containing 50 mL of PDB liquid medium; the fermentation culture was oscillated at 28 °C and 150 r/min for 7d. The supernatant was centrifuged at 4 °C and 12000 r/min for 10 min and

then filtered through a 0.22 μm sterile filter membrane, and the fermentation broth was obtained as the metabolites of the endophytic fungi. The blank drug-sensitive paper was placed in the bacterial plate, and each plate was inoculated with 3 pieces of filter paper at equal distance, and 15μL of fermentation broth (endophytic fungal metabolites) was added to each piece of filter paper dropwise by pipette gun; 3 groups of replicates were set up for each endophytic fungal metabolites, and the blank drug-sensitive paper was used as the blank control, and the culture was incubated at a constant temperature of 28 °C, and whether there was any circle of inhibition was observed and the size of the circle of inhibition was measured after 3 d, and the mean value was taken for calculation.

Determination of bacteriostatic activity of endophytic bacterial metabolites

Pick a small amount of pure endophytes, inoculate them into fresh LB medium by plate streaking method, activate and cultivate them at 30 ℃ for 12~16 h. Pick the activated single colony and transfer it to LB liquid medium, incubate it at 30℃ and 230 r/min overnight; take 2 mL of the seed solution into 50 mL of the newly formulated LB liquid medium, incubate it at 30℃and 180 r/min for 7d, and then centrifuge the supernatant after centrifugation for 5 min at 8000 r/min, and filter the fermentation broth obtained by filtration with 0.22 μm sterile filter membrane to remove bacteria. After centrifugation, the supernatant was filtered with a 0.22 μm sterile membrane to remove bacteria, and the fermentation broth obtained from the filtration was endophytic bacterial metabolites. Determination of bacteriostatic activity of endophytic bacterial metabolites was the same as 1.9.2.

Results and analysis

Results of an orthogonal test on seed disinfection of Panax japonicus

The results of the effects of different disinfection time and disinfectant concentration on the disinfection effect of *Panax japonicus* seeds showed that 75%

alcohol disinfection for 30 s and 1% $HgCl_2$ disinfection for 9 min resulted in the lowest contamination rate of 3.33% (Table 1), and the analysis of extreme variance (ANOVA) indicated that the three factors had the greatest effect on the disinfection effect of *Panax japonicus* seeds as $HgCl_2$, followed by disinfection time and finally 75% alcohol; ANOVA pointed out that the differences between the set ANOVA indicated that the differences among 75% alcohol, $HgCl_2$ and disinfection time on the contamination rate of *Panax japonicus* seeds were not significant at the statistical level within the set gradient range (Table 2). However, it was obtained that $HgCl_2$ (%) > time (min) > 75% alcohol affected seed disinfection in the order of priority (Table 3).

Table 1 Results of the orthogonal test of seed disinfection of *Panax japonicus.japonicus*.

deal with	factor			Results statistics		
	75% alcohol (s)	$HgCl_2$ (%)	Time (min)	Number of inoculations	contami nation	Contamination rate (%)
1	30	0.1	3	30	10	33.33
2	30	0.5	6	30	2	6.67
3	30	1.0	9	30	1	3.33
4	60	0.1	6	30	18	60.00
5	60	0.5	9	30	4	13.33
6	60	1.0	3	30	6	20.00
7	90	0.1	9	30	7	23.33
8	90	0.5	3	30	14	46.67
9	90	1.0	6	30	4	13.33

Table 2 Analysis of variance (ANOVA) of the factors affecting seed disinfection of *Panax japonicus*.

	Type III sum of squares	Degree of freedom	Mean square	F	Signific ance
alcohol	466.667	2	233.333	0.677	0.596
$HgCl_2$	1088.844	2	544.422	1.58	0.388
time	622.444	2	311.222	0.903	0.525
inaccuracies	688.978	2	344.489		

Table 3Extreme variance analysis of factors affecting seed disinfection of *Panax japonicus*.

Item	75% alcohol (s)	$HgCl_2$ (%)	Time (min)
**K1**	43.33	116.67	100.00
**K2**	93.33	66.67	80.00
**K3**	83.33	36.67	40.00
**k1**	14.44	38.89	33.33
**k2**	31.11	22.22	26.67
**k3**	27.78	12.22	13.33
**R**	16.67	26.67	20.00
Order of priority	$HgCl_2$ (%) > time (min) > 75% alcohol		

Isolation and purification of endophytic bacteria from the seeds of *Panax japonicus*

Seven endophytic fungal colonies, named *Pj*Z1, *Pj*Z2, *Pj*Z3, *Pj*Z4, *Pj*Z5, *Pj*Z6, *Pj*Z7, and three endophytic bacterial colonies, named *Pj*X1, *Pj*X2, and *Pj*X3, were isolated from the seeds of *Panax japonicus.*The morphology of all three endophytic bacteria was round, with neat edges and slightly raised centers, the colonies were creamy white, and the results of Gram staining were negative(Table4), the morphology of the fungi is described in Table5.

Fig.1 Characterization of colonies of the endophytic fungi *Pj*Z1-*Pj*Z7 in the seeds of *Panax japonicus*

Fig.2 Characterization of colonies and Gram staining results of endophytic bacteria *Pj*X1, *Pj*X2, and *Pj*X3 from seeds of *Panax japonicus*

Table 4 Morphology and Gram staining of endophytic bacteria in the seeds of

Panax japonicus

Endophytic bacteria	Colony characteristics	Gram stain
*Pj*X1	Rounded, with neat margins and a slightly raised center, the colonies are creamy white	negatives
*Pj*X2	Rounded, with neat margins and a slightly raised center, the colonies are creamy white	negatives
*Pj*X3	Rounded, with neat margins and a slightly raised center, the colonies are creamy white	negatives

Table 5 Characteristics of endophytic fungal colonies in the seeds of *Panax japonicus.*

Endophytic fungi	Appearance	Microscopic form
*Pj*Z1	Mycelium abundant, aerial hyphae well-developed; colonies raised, tomentose, white on the front, orange on the back, with goose-yellow margins.	Mycelium septate, branched; spores green,rounded;broom-like spore-producing structures.
*Pj*Z2	Mycelium fewer, aerial hyphae well developed; colonies raised, tomentose, adaxial margin yellow, middle pink, abaxial light yellow-brown, margin goose-yellow.	Mycelium septate, branched; spores green,rounded;broom-like spore-producing structures.
*Pj*Z3	Mycelium abundant, aerial hyphae well-developed; colonies raised, tomentose, white adaxially, light yellow abaxially.	Mycelium septate, branched; spores green,rounded;broom-like spore-producing structures.
*Pj*Z4	Mycelium abundant, aerial hyphae well-developed; colonies raised, tomentose, gray-yellow adaxially, brown abaxially, with goose-yellow margins.	Mycelium septate, branched; spores colorless,rounded;broom-like spore-producing structures.
*Pj*Z5	Mycelium abundant, aerial hyphae well developed; colonies raised, cottony, white on the front, goose-yellow on the back.	Mycelium septate, branched; spores green,rounded;broom-like spore-producing structures.
*Pj*Z6	Mycelium fewer, aerial hyphae well developed; colonies raised, downy, pink on the front, light orange on the back.	Mycelium septate, branched; spores green,rounded;broom-like spore-producing structures.
*Pj*Z7	Mycelium abundant, aerial hyphae well-developed; colonies raised, tomentose, grayish-white adaxially, brown in the middle of the abaxial surface, grayish-brown at the edges.	Mycelium septate, branched; spores colorless,rounded;broom-like spore-producing structures.

Molecular biological identification and homology analysis of endophytic bacteria

ITS sequence analysis indicated that the sequence lengths of seven endophytic

fungi were *Pj*Z1 511bp, *Pj*Z2 459bp,*Pj*Z3 502bp,*Pj*Z4 489bp,*Pj*Z5 607bp,*Pj*Z6 519bp, and *Pj*Z7 512bp, and the size of the target bands amplified by PCR using the universal primers ITS1 and ITS4 (Fig.3) was in agreement with the sequencing results. The 16SrDNA sequence analysis indicated that the sequence lengths of the three endophytic bacteria were *Pj*X1 1413bp, *Pj*X2 1400bp, and *Pj*X3 1416bp, respectively, and the size of the target bands amplified by PCR using the universal primers 27F and 1492R (Fig.4) was consistent with the sequencing results.BLAST search was performed at NCBI and a Utilization of the software MEGA11 phylogenetic tree was constructed (Fig.5), which revealed that the sequence similarity between endophytic fungus *Pj*Z1 and accession number JX827458.1 was 100% and identified as *Fusarium tricinctum*;*Pj*Z2 and accession number MT601889.1 had 100% sequence similarity and identified as *Fusarium reticulatum*; *Pj*Z3 showed 100% sequence similarity to accession number JX401981.1 and was identified as *Fusarium tricinctum*; *Pj*Z4 showed 100% sequence similarity to accession number OR671459.1 and was identified as *Fusarium tricinctum*;*Pj*Z5 showed 96.67% sequence similarity with accession number OR543773.1 and was identified as *Fusarium tricinctum*;*Pj*Z6 showed 100% sequence similarity with accession number LT746261.1 and was identified as *Fusarium sarcochroum*; *Pj*Z7 showed 100% sequence similarity with accession number LT746261.1 with 100% sequence similarity, identified as Streptomyces *alternaria alternata*. endophytic bacteria *Pj*X1 with accession number JQ864378.1 with 100% sequence similarity, identified as *Enterobacteriaceae bacterium*,*Pj*X2 with accession number PQ097710.1 with 99.93% sequence similarity and identified as *Pseudomonas aeruginosa*, *Pj*X3 with 99.79% sequence similarity to accession number JX067703.1 and identified as *Enterobacteriaceae* bacterium.

Fig.3 PCR identification of endophytic fungi in the seeds of *Panax japonicus*.

Marker:DNA Marker DL2000; 1-3 for *Pj*Z1, 4-6 for *Pj*Z2, 7-9 for *Pj*Z3, 10-12 for *Pj*Z4, 13-15 for *Pj*Z5, 16-18 for *Pj*Z6, 19-21 for *Pj*Z7

Fig.4 PCR identification of endophytic bacteria in the seeds of *Panax japonicus*.

Marker:DNA Marker DL2000; 1-3 for *Pj*X1, 4-6 for *Pj*X2, 7-9 for *Pj*X3

Fig5 Phylogenetic tree of endophytes

a. Phylogenetic tree constructed from *Pj*Z1-*Pj*Z7 rDNA-ITS sequences of the endophytic fungi from the seeds of *Panax japonicus*,

b. Phylogenetic tree constructed from the rDNA sequences of endophytic bacteria *Pj*X1-*Pj*X3 in the seeds of *Panax japonicus*

Determination of bacteriostatic activity of endophytic fungal bacteria

Determination of bacteriostatic activity were carried out on seven strains of endophytic fungi isolated with Escherichia coli, Staphylococcus aureus and Bacillus subtilis, and the results showed that (Table 6), strain *Pj*Z4 had inhibitory activity against Escherichia coli, but the inhibitory activity was weak, with an average inhibitory circle diameter of 10.48 mm, and the remaining six strains of the fungi did not have any inhibitory activity against E. coli; the seven strains of the endophytic fungi strains did not have any inhibitory activity against Staphylococcus aureus; Strain *Pj*Z3, *Pj*Z4, *Pj*Z5, *Pj*Z7 had bacteriostatic activity against B.subtilis, strain *Pj*Z3 had the strongest bacteriostatic activity against B. subtilis, with an average inhibitory circle diameter of 23.53mm, strain *Pj*Z4 had the weakest inhibitory activity against B. subtilis, with an average inhibitory circle diameter of 13.69mm, and the average inhibitory circle diameters of strains *Pj*Z5 and *Pj*Z7 were respectively 16.80mm, 17.97mm, strains *Pj*Z1, *Pj*Z2 and *Pj*Z6 had no inhibitory activity against B. subtilis, and no inhibitory circle was produced in the control blank drug-sensitive paper sheet (Figure 6).

Fig. 6 Inhibitory activity of endophytic fungi from the seeds of *Panax japonicus* against three pathogenic bacteria

A、B and C are blank controls for the three pathogens; D, *Pj*3/Bacillus subtilis; E, *Pj*4/E. coli; F, *Pj*4/Bacillus subtilis; G, *Pj*5/Bacillus subtilis; and H, *Pj*7/Bacillus subtilis.

Table 6 Inhibitory activity of endophytic fungi from the seeds of *Panax japonicus* against three pathogenic bacteria

Strain number	Escherichia coli (E. coli)	Staphylococcus aureus	Bacillus subtilis
	Diameter of inhibition circle/ mm		
*Pj*Z1	-	-	-
*Pj*Z2	-	-	-
*Pj*Z3	-	-	23.53
*Pj*Z4	10.48	-	13.69
*Pj*Z5	-	-	16.80
*Pj*Z6	-	-	-
*Pj*Z7	-	-	17.97

Bacteriostatic activity of endophyte metabolites

Antagonistic assay of the metabolites of 7 strains of endophytic fungi and 3 strains of bacteria isolated against Escherichia coli, Staphylococcus aureus and Bacillus subtilis showed that (Table7), the metabolites of the 7 strains of endophytic fungi had no inhibitory activity against Escherichia coli, Staphylococcus aureus and Bacillus subtilis, and there was no inhibitory circle on the control blank drug-sensitive paper sheet as well.

Antagonistic assay of the metabolites of the three isolated endophytic bacteria with E. coli, S. aureus and B. subtilis revealed that the metabolites of the three endophytic bacteria had no bacteriostatic activity against E. coli, S. aureus and B. subtilis.(Table8)

Table 7 Inhibitory activity of endophytic fungal metabolites from the seeds of *Panax japonicus*

Strain number	Escherichia coli (E. coli)	Staphylococcus aureus	Bacillus subtilis
*Pj*Z1	—	—	—
*Pj*Z2	—	—	—
*Pj*Z3	—	—	—
*Pj*Z4	—	—	—
*Pj*Z5	—	—	—
*Pj*Z6	—	—	—
*Pj*Z7	—	—	—

Table 8 Inhibitory activity of endophytic fungal metabolites from the seeds of *Panax japonicus*

Strain number	Escherichia coli (E. coli)	Staphylococcus aureus	Bacillus subtilis

PjZ1	—	—	—
PjZ2	—	—	—
PjZ3	—	—	—
PjZ4	—	—	—
PjZ5	—	—	—
PjZ6	—	—	—
PjZ7	—	—	—

Discussion

In recent years, the germplasm resources of *Panax japonicus* have been endangered, and the conservation of the germplasm resources of *Panax japonicus* has become a key issue to be solved. In the process of artificial breeding, we found that the seeds of *Panax japonicus* were difficult to germinate and the seeds of *Panax japonicus* were difficult to germinate., studies have shown that different plant seed endophytes can potentially increase the productivity of host plants[12]. However, the isolation and identification of endophytic fungi from the seeds of *Panax japonicus* have not been reported.In this study, seven strains of endophytic fungi were isolated from the seeds of *Panax japonicus*, among which six strains belonged to *Fusarium,*and one strain belonged to *Alternaria alternata* The two genera of fungi had almost no inhibitory effect on Escherichia coli and Staphylococcus aureus, and had inhibitory activity on bacillus cereus but the inhibitory effect was not obvious, and the metabolites of the two genera had no inhibitory activity on the indicator organisms. The metabolites of the two genera of fungi had no bacteriostatic activity on the indicator bacteria, indicating that the two genera of fungi were not the dominant species of endophytic fungi in the seeds of *Panax japonicus*. And many studies pointed out that *Fusarium*. and *Alternaria alternata* are pathogenic fungi that cause plant diseases, such as Yang isolation and identification of pathogenic fungi on naturally-occurring *Passiflora edulis*, and found that the pathogenic fungi that cause post-harvest *Passiflora edulis* diseases belong to *Fusarium* spp. and *Streptomyces* .[13] Zhong determined the pathogenicity of different strains of *Fusarium* species on *Nicotiana tabacum*, and showed that the pathogenic

fungi of *Fusarium* root rot of *Nicotiana tabacum* were mainly shared by *Fusarium*., and 40% of imipramine EW had an inhibitory effect on this pathogen[14].Chen isolated and identified the pathogenic bacteria from the leaves of the diseased plants of *Pseudocercospora variicola*, and the results showed that the pathogen of *Pseudocercospora variicola* was *Alternaria alternata.*[15].All these studies confirmed that *Fusarium*. and *Alternaria alternata*. are not dominant species in plants.still ,both of them cause different diseases and inhibit the normal growth and development of plants in various degrees.Therefore, it is likely that the difficulty in seed germination of *Panax japonicus* is due to the action of these two endophytic fungi, and further research is needed to find antibiotics to inhibit the growth of this endophytic fungus in order to solve the problem of the difficulty in seed germination of *Panax japonicus*.

In this study, three strains of endophytic bacteria were also isolated from the seeds of *Panax japonicus*, two of which belonged to *Enterobacteriaceae* and one belonged to *Pseudomonas aeruginosa*, and the metabolites of the two genera of bacteria had no inhibitory activity against the indicator bacteria, which may be due to the high concentration of the indicator bacteria or the low activity of the metabolites. Some studies have shown that Enterobacteriaceae genus has a better effect in promoting plant growth, such as Zhao isolated endophytic Enterobacteriaceae from the *Paspalum vaginatum* as a material to explore the germination of dogbane seeds and salt tolerance after ping, the results of the seed germination rate and salt tolerance after ping has been improved.[16] Some scholars isolated endophytic bacteria from the root system of cucumber, and found that Enterobacteriaceae and Pseudomonas spp. played an important role in the control of cucumber wilt disease[17].Ma initially identified eight strains of bacteria isolated from plant roots and investigated the growth-promoting effects of endophytic bacteria on ryegrass, which showed that all eight strains of bacteria belonged to *Pseudomonas aeruginosa*. and could effectively promote the growth and development of *Lolium perenne*[18].The results showed that all eight strains of bacteria belonged to Pseudomonas spp. From the results of many studies, it can be inferred that *Enterobacteriaceae* and *Pseudomonas aeruginosa*. are the dominant strains in promoting plant growth and development, therefore, further investigation can

be carried out in the future seed germination and disease control of *Panax japonicus*.

Conclusion

The orthogonal experiment screened out the optimal disinfection conditions of *Panax japonicus* seeds as 75% alcohol disinfection for 30s and 1% $HgCl_2$ disinfection for 9min, which had the lowest contamination rate of 3.33%. Seven strains of endophytic fungi *Pj*Z1, *Pj*Z2, *Pj*Z3, *Pj*Z4, *Pj*Z5, *Pj*Z6, *Pj*Z7 and three strains of *endophytic bacteria* *Pj*X1, *Pj*X2, *Pj*X3 were isolated and screened from the seeds of *Panax japonicus*, and the strains of *Pj*Z1, *Pj*Z3, *Pj*Z4, *Pj*Z5 were identified as the fungi of the *Fusarium* genus, and the strains of *Pj*Z2 and *Pj*Z4 were identified as the fungi of the *Fusarium* genus. *Fusarium reticulatum*, *Pj*Z6 is *Fusarium sarcochroum*, *Pj*Z7 is Alternaria alternata; *Pj*X1 and *Pj*X3 are *Enterobacteriaceae bacterium*, and *Pj*X2 is *Pseudomonas aeruginosa*. The results of inhibition experiments showed that only *Pj*Z4 had inhibitory activity against E. coli, *Pj*Z3, *Pj*Z4, *Pj*Z5, *Pj*Z7 had inhibitory activity against Bacillus subtilis, and none of the 7 endophytic fungal strains had inhibitory activity against Staphylococcus aureus, and the metabolites of the 10 endophytic strains of endophytic fungi were antagonized with E. coli, Staphylococcus aureus, and Bacillus subtilis. The experiment showed that the metabolites of 10 strains of endophytic bacteria had no bacteriostatic activity against Escherichia coli, Staphylococcus aureus and Bacillus subtilis. After preliminary judgment, the isolated *Fusarium spp.* and *Alternaria alternata*. are not the dominant species in the growth and development process of *Panax japonicus*, and it can be inferred that the difficulty in seed germination of bamboo ginseng may be caused by the growth of these two genera of fungi inhibiting the

synthesis of growth hormone by the seeds during the seed germination process. The isolated *Enterobacteriaceae bacterium* and *Pseudomonas spp*. are the dominant species in promoting plant growth and development, so these two genera of bacteria can be utilized in the cultivation process of *Panax japonicus* to promote its growth and development.The endophytes isolated in this study provide a theoretical basis for the screening of endophyte antagonist strains of *Panax japonicus* and for seed germination and disease control of *Panax japonicus*.

Acknowledgements

We would like to thank Professor Lai Zhang for his careful guidance of this study ,School of Life Science, Guizhou Normal University and the Guizhou Provincial Innovation Center for Mountainous and Efficient Agriculture for its strong support.

We thank the editors and reviewers for their hard work on our papers.

Reference

[1] ZHOU Jing, FU Xian Yun, CAI San Jin, YUAN Ding, MEI Zhigang, FENG Zhitao. [2]Research progress on chemical constituents and pharmacological effects of *Panax japonicus* and predictive analysis on quality markers [J]. Chinese Herbal Medicine,2021,52(09):2819-2830

[2] SHEN Peiyao, ZHANG Ye, LI Yuqin, YAN Jianye, WANG Yuanqing. [3]Research progress on biological activity and quality control of *Panacis Japonici Rhizoma*[J]. Food and Machinery,2021,37(11):211-220.

[3] GUO Zhe, FENG Zhitao, ZHANG Haoran, YAN Lan, LIANG Mingge, MEI Zhigang, CAI Sanjin. Progress of research on *Panax japonicus* and its preparation for the treatment of rheumatoid arthritis[J]. Chinese Materia Medica,2019,42(04):941-944.

[4] LI Chunyan, ZHANG Jie, LI Jinping, et al. Research on Chemical Constituents and Biological Activities of Rhizoma of *Panax japonicus*[J]. Introduction to Traditional Chinese Medicine,2012,18(04):68-71.DOI:10.13862/j.cnki.cn43-1446/r.2012.04.050.

[5] YANG Fajian,DING Guoyu,WANG Hongyun,et al. Technical points of seed multiplication of the rare plant Bamboo Ginseng[J]. Southern Agriculture,2020,14(29):19-20. DOI:10.19415/j.cnki.1673-890x.2020.29.010.

[6] CHENG Longyuan, ZHANG Guohui, SUN Yan, et al. Progress of medicinal plant-endophyte-inter-root microbial interactions[J]. Chinese Herbal Medicine,2024,55(15):5264-5273.

[7] Liang E.Endophytic Bacterial Diversity and Cloning＆BioinformaticsAnalysis of Squalene epoxidase(SE)Gene in *Panax japonicusC.A.Meyer*[D]. Guizhou Normal University,2020.DOI:10.27048/d.cnki.ggzsu.2020.000068.

[8] ZHU Lili,ZHANG Yemeng,LI Wancai,et al. Comprehensive evaluation of the effects of different disinfectants on surface disinfection and germination of quinoa seeds[J]. Journal of Biology,2023,40(4):83-89. DOI:10.3969/j.issn.2

095-1736.2023.04.083.

[9] ZHANG Yubai, WEN Xiangshui, WANG Yumeng, et al. Isolation and characterization of endophytic fungi from the seeds of Fructus schisandrae chinensis and their fungicidal properties[J]. Chinese Materia Medica,2020,43(5):1087-1091. DOI:10.13863/j.issn1001-4454.2020.05.008.

[10] Wei, Jingchao. Handbook of fungal identification [M]. Shanghai: Shanghai Science and Technology Press, 1979.09.

[11] Dong Xiuzhu, Cai Miaoying, et al. eds. Handbook of Systematic Identification of Common Bacteria [M]. Beijing: Science Press, 2001.02.

[12] Liang Danni. Mechanisms of Seed Endophytic BacteriaRegulating Nitrogen Transformation[D]. Lanzhou University,2023.DOI:10.27204/d.cnki.glzhu.2023.004000.

[13] YANG Dingzhao,SHUAI Liang,TANG Suizhi,et al. Isolation and identification of passion fruit derived pathogenic bacteria and preliminary study on inhibitory activity of bactericidal agents[J/OL]. Food and Fermentation Industry,1-12[2024-08-19].https://doi.org/10.13995/j.cnki.11-1802/ts.040144.

[14] ZHONG Jie, QIU Zelan, LIU Shida, et al. Pathogen Identification of Tobacco Root Rot Disease in Hunan Province and Screening of Control Fungicides[J]. China Tobacco Science,2024,45(03):51-59+67.DOI:10.13496/j.issn.1007-5119.2024.03.008.

[15] CHEN Cun,LIU Yan,ZHAO Yuyang,et al. Isolation and characterization of the pathogenic bacteria of Verticillium verticillioides in Paeonia lactiflora[J/OL]. Chinese Materia Medica,2024,(07):1630-1633[2024-08-19].https://doi.org/10.13863/j.issn1001-4454.2024.07.005.

[16] ZHAO Xintong,CHEN Xiaodong,LI Ziji,et al. An evaluation of the effects of the plant endophyte Enterobacter on the salt tolerance of bermudagrass [J]. Journal of Grass Industry,2021,30(09):127-136.

[17] Yu-Lu Zhang 1,Xiao-Jing Guo 1,Xin Huang 1 2,Rong-Jun Guo 1,Xiao-Hong Lu 1,Shi-Dong Li 1,Hao Zhang 2.The Co-Association of Enterobacteriaceae and Pseudomonas with Specific Resistant Cucumber against Fusarium W

ilt Disease[J].Biology.2023,Vol.12(No.2):143.
[18]MA Yachun,YAO Tuo,LEI Yang,et al. Screening of pseudomonas inorganic phosphate and its effect on ryegrass growth[J]. Grassland and Turf,2024,44 (03):101-107.DOI:10.13817/j.cnki.cyycp.2024.03.013.

Supporting Information

S1_raw_images

Response to Reviewers

Dear editors and reviewers,

Thank you for your comments on the title of our manuscript 'Isolation, identification and antibacterial activity of endophyte from the seeds of *Panax japonicus* '. Your comments will help us to revise and improve the manuscript, and will also play an important guiding role in our future research in the Panax japonicus or other fields. We are very grateful to you for your comments and comments on the content of our manuscripts, especially for the questions we have pointed out in words. We have carefully studied these comments and tried to modify the manuscript, hoping to get your approval. Revisions are marked in red in the manuscript. The following is a response to your comments.

Editor

1.Please ensure that your manuscript meets PLOS ONE's style requirements, including thosefor file naming.

Response: Dear Editor, thank you for your reminder that we have modified the format and naming according to the journal requirements.

2.Please state what role the funders took in the study.

Response:Thanks to the editors for being so careful, we have clarified in the Cover letter: “The above four project funds provide financial support and project guidance for this research.”

3.We note that you have provided funding information that is currently declared in your Funding Statement. However, funding information should not appear in the Acknowledgments section or other areas of your manuscript. We will only publish funding information present in the Funding Statement section of the online submission form.Please remove any funding-related text from the manuscript and let us know how you would like to update your Funding Statement.

Response:Thanks for the editor's reminder and suggestion, we have made modifications, deleted the text related to funding from the manuscript, and included the funding information in the Cover letter.

4.When completing the data availability statement of the submission form, you

indicated that you will make your data available on acceptance. We strongly recommend all authors decide on a data sharing plan before acceptance, as the process can be lengthy and hold up publication timelines. Please note that, though access restrictions are acceptable now, your entire data will need to be made freely accessible if your manuscript is accepted for publication. This policy applies to all data except where public deposition would breach compliance with the protocol approved by your research ethics board. If you are unable to adhere to our open data policy,please kindly revise your statement to explain your reasoning and we will seek the editor's input on an exemption. Please be assured that, once you have provided your new statement, the assessment of your exemption will not hold up the peer review process.

Response:Thanks for the editor's suggestion, we have started to prepare the work of data uploading and data sharing, and we promise to do our best to complete the work before the manuscript is accepted.

5.PLOS ONE now requires that authors provide the original uncropped and unadjusted images underlying all blot or gel results reported in a submission's figures or Supporting Information Files.In your cover letter, please note whether your blot/gel image data are in Supporting Informationor posted at a public data repository, provide the repository URL if relevant, and provide specificdetails as to which raw blot/gel images, if any, are not available. Email us at plosone@plos.org if you have any questions.

Response:Thanks for the editors' valuable feedback and suggestions on the chart information. We have put the unadjusted chart into the file separately and explained it in the Cover letter: we will agree that blot/gel image data are in Supporting Information or posted at a public data repository.

6.Please remove your figures from within your manuscript file, leaving only the individual TIFF/EPS image files, uploaded separately ,and please include your tables as part of your main manuscript and remove the individual files.

Response:Thanks to the careful reminding of the editor, we have uploaded the single TIFF/EPS image file and deleted the chart from the manuscript, and the single table file has also been deleted.

Reviewer 1

Abstract (Lines 39-65):

• Line 40: Consider stating the specific aim of this study more clearly, such as: "The aim of this study was to isolate..."

Response:We agree with the reviewer on this point, and we have revised it in the manuscript: " The purpose of this study is to isolate and identify endophytic bacteria from the seeds of *Panax japonicus*, finding a dominant strain that favors seed germination in *Panax japonicus* and to provide theoretical reference for further research on the effect of endophytic bacteria on seed germination. "

• Line 48: It is confusing to read "Seven endophytic fungi strains were isolated" immediately followed by mentioning that "*Pj*Z1, *Pj*Z3, *Pj*Z4 and *Pj*Z5 were identified as Fusarium". This makes it appear that only these four were fungi and not all 7 strains. You should rephrase this to clarify
that ALL 7 are fungal.

Response:We thank the reviewer for making this important point, which we have elaborated in the manuscript: "Seven endophytic fungi strains were isolated from the seeds of *Panax japonicus*, named *Pj*Z1, *Pj*Z2, *Pj*Z3, *Pj*Z4, *Pj*Z5, *Pj*Z6, and *Pj*Z7, respectively. Four strains belonged to the genus *Fusarium tricinctum*, one to *Fusarium reticulatum*, one to *Fusarium sarcochroum*, and one to *Alternaria alternata"*

• Line 55: Instead of "Only PjZ4 had antibacterial activity", it may be more appropriate to say,"Among the isolates, only PjZ4 showed significant antibacterial activity...". This highlights that the other fungal isolates did have some inhibition on Bacillus subtilis, even if not significant.

Response:We thank the reviewer for pointing out a more appropriate statement for us, and we have readjusted it: "Among the endophytic fungal strains isolated, only PjZ4 showed significant bacteriostatic activity against Escherichia coli. "

• Line 58: Conclusion: It is advised to have a stronger conclusion. For instance, the authors could summarize the main findings, including the genus level of the identified strains, and highlight the significance of their study for future research.

Response:Thanks to the reviewer for his valuable suggestions. In view of the suggestions, we have made a new explanation after careful consideration:“In this study, we investigated the method of bamboo ginseng seed disinfection, and the results showed that 75% alcohol disinfection for 30s and 1% $HgCl_2$ disinfection for 9min resulted in the lowest contamination rate of 3.33%, in which the greatest influence on the seed disinfection effect was the concentration of $HgCl_2$. Meanwhile, 11 endophyte species of *Panax japonicus* seeds were isolated and identified, *Fusarium spp*. and *Alternaria alternata*. were not the dominant species in the growth and development process of *Panax japonicus* while *Enterobacteriaceae* and *Pseudomonas sp.* were the dominant species in the promotion of plant growth and development, and provides a theoretical reference for further research on the biological functions and active substances of endophytic fungi in the seeds of *Panax japonicus*.”

• Line 63: The keywords could be improved by making them more specific. For example, replace "Antibacterial activity" with "Endophytic antibacterial activity".

Response:Thanks for your helpful suggestions, we have updated the keyword "Antibacterial activity" to "Endophytic antibacterial activity"

Introduction (Lines 66-81):

• Line 68: The phrase "also known as Disporopsis fuscopicta Hance" is an unnecessary detail here as it is not the same genus.

Response:We appreciate your reminder that we have removed the word from the manuscript.

• Line 70: Should add citations to support that "Pharmacological studies have shown that Panax japonicus..."

Response:We are grateful for your reminder that in the manuscript, our citation for this part reads [2-4]:[2]SHEN Peiyao, ZHANG Ye, LI Yuqin, YAN Jianye, WANG Yuanqing. Research progress on biological activity and quality control of Panacis Japonici Rhizoma[J]. Food and Machinery,2021,37(11):211-220. [3]GUO Zhe, FENG Zhitao, ZHANG Haoran, YAN Lan, LIANG Mingge, MEI Zhigang, CAI Sanjin. Progress of research on Panax japonicus and its preparation for the treatment of

rheumatoid arthritis[J]. Chinese Materia Medica,2019,42(04):941-944. [4]LI Chunyan, ZHANG Jie, LI Jinping, et al. Research on Chemical Constituents and Biological Activities of Rhizoma of Panax japonicus[J]. Introduction to Traditional Chinese Medicine,2012,18(04):68-71.DOI:10.13862/j.cnki.cn43-1446/r.2012.04.050.

• Line 75: The statement regarding lack of reports on endophytic fungi/bacteria from seeds is good, but there needs to be something to indicate why this is valuable. For example, why are seeds and what importance is this to *P. japonicus*? And Line 77-80: The end of the introduction, there should be something to emphasize the biological functions of the bacteria isolated.

Response:We would like to thank the reviewer for pointing out this problem. The clarification of this problem will make the purpose of this paper more clear. Therefore, we have further elaborated this part in the manuscript: "Because of the high efficacy and high price of the rare wild Chinese herbal medicine, it is almost difficult to find wild resources of *Panax japonicus* in the wild, so the artificial propagation of Panax japonicus seeds is urgent.Therefore, the selection of *Panax japonicus* seeds, leaves and stems for seedling propagation is a better choice of explants. It is noteworthy that the seeds of *Panax japonicus* have the phenomenon of after-ripening, and the contamination rate is high during the germination process of histoculture, which makes the germination difficult. Therefore, we carried out further exploration. It has been shown that endophytes of medicinal plants are important for promoting the growth of medicinal plants, improving the quality of medicinal plants, and eliminating the barriers of continuous cultivation."

Materials and Methods (Lines 82-162):

• Line 86: Provide the geographic coordinates or location details of the experimental site.

Response:We would like to thank the reviewer for suggesting that our detailed address is"Guizhou Innovation Center of Mountain Specialty and Efficient Agriculture" of Anshun College, Anshun City, Guizhou Province, China, has been presented in the manuscript.

• Line 90: The composition of NA (Nutrient Agar) should be described better. The

recipe is given as "Peptone 10g, Beef Paste 3g, Sodium Chloride 5g, Agar 20g, distilled water 1000mL, pH:7.2." It would be better practice to say "Beef Extract" instead of "Beef Paste", as well as identify if peptone is tryptone or another form.

Response:We would like to thank the reviewers for their careful comments on this and we have corrected "Beef Extract" to" Beef paste".

• Line 103: Provide a citation or additional details to justify the use of this orthogonal testing method.

Response:Thank you very much for your professional advice and we have added relevant citations in this part of the manuscript:[8]ZHU Lili,ZHANG Yemeng,LI Wancai,et al. Comprehensive evaluation of the effects of different disinfectants on surface disinfection and germination of quinoa seeds[J]. Journal of Biology,2023,40(4):83-89. DOI:10.3969/j.issn.2095-1736.2023.04.083.

• Line 107-109: It is difficult to follow that section. Re-word and clarify that after disinfection, the seeds were plated to analyze contamination rate before proceeding with isolation.

Response:We thank the reviewers for their important comments on this, which we have rephrased in the manuscript:“Prior to the isolation of endophytes from the seeds of *Panax japonicus*, the seeds of *Panax japonicus* were sterilized under different conditions and the contamination rate under different sterilization conditions were analyzed.”

• Line 117: Clarify why the seed is cut in half before plating.

Response:Thank you very much for your professional advice and we have added relevant citations in this part of the manuscript:[9]ZHANG Yubai, WEN Xiangshui, WANG Yumeng, et al. Isolation and characterization of endophytic fungi from the seeds of Fructus schisandrae chinensis and their fungicidal properties[J]. Chinese Materia Medica,2020,43(5):1087-1091. DOI:10.13863/j.issn1001-4454.2020.05.008.

• Line 119: What is meant by "seed preservation" here? It is better to say for "preservation of the isolated strains"

Response:We thank the reviewers for their very meticulous comments, which we have reorganized: “After the growth of mycelium, pick the mycelium for repeated isolation

and purification for 2-3 times for preservation of the isolated strains."

• Line 127-136: The morphological observation section lacks clarity. How many samples were observed per isolate? Were observations done in replicates? More detail on how results were "recorded" should be included.

Response:Thanks to the reviewer's constructive comments on this, we have re-elaborated in the manuscript: "Pick the purified fungal hyphae and make them into clinical slices, observe and record (hyphae color, hyphae morphology, spore morphology, etc.) through an optical microscope, and refer to "Fungal Identification Manual".etc. for preliminary identification of fungi. (Three strains on each medium for each strain and three strains on each medium for observation and identification)."

• Line 137-139: The DNA extraction procedure needs to be described with more detail: What is the kit that was used? Also, it is better to use "according to the manufacture's instructions" rather than just stating "according to the instructions".

Response: Thanks to the reviewer for alerting us to this problem, which we have reflected in the manuscript:"Endophytic fungal DNA was extracted according to the instructions of the fungal DNA extraction kit (Solarbio), and endophytic bacterial DNA was extracted according to the instructions of the bacterial DNA extraction kit. Subsequently, PCR amplification was performed using the DNA of each endophytic bacterium as a template, respectively."

• Line 144: The reaction volume and composition is confusing. It would be better to say "a 50uL reaction volume containing..." instead.

Response: Thanks to the reviewer's attention to the following details, we have corrected the statement:"Endophytic fungi were amplified by PCR using the universal primers of fungi, ITS1 (5'-TCCGTAGGTGAACCTGCGG-3') and ITS4 (5'-TCCTCCGCTTATTGATATGC-3') as primers, and the total volume of the PCR reaction system was 50μL, containing:4μL of DNA template, 2μL of each of ITS1 and ITS4, and 2×MasterMix 25μL, and add ddH_2O 17μL."

• Line 151: The statement about the primer name is inconsistent, the previous section mentioned primer ITS1 and ITS4 but now are described as "primers 27 F (5'-AGAGTTTGATCCTGGCTCAG-3') and 1492R (5'-TACGGCTACCTTGTTA

CGAC".

Response: Thanks to the reviewer for raising this question. The inconsistent names of primers are due to the fact that the endophytic bacteria isolated in our paper include fungi and bacteria, and the common primers of the two bacteria are different, so the names and sequences of the two primers appear in the paper.

• Line 157: What percentage of agarose gel was used in gel electrophoresis?

Response:Thanks for the reviewer's attention to this issue: In the manuscript we mentioned that the percentage of agarose gel used was 1%.

• Line 166: Please add more detail about the selection of pure isolates. For instance, whether single colonies were used to prepare the pure isolates,and Line 167: What does “adjusted to OD600=0.2 in 0.85% saline”means? Also, how was the 0.2 OD reading achieved?

Response:Thanks to the reviewers, we have described the process more clearly in the manuscript and updated the text:“The pure indicator strains (Staphylococcus aureus, Bacillus subtilis, Escherichia coli) were picked and inoculated onto LB medium by plate streaking method for overnight cultivation; Pick single colonies on LB medium again for plate streaking overnight culture; pick single colonies to 0.85% saline to prepare the bacterial solution, adjust the concentration of the bacterial solution and measured by UV-visible spectrophotometer (UV-5500, Shanghai, China), until OD600 = 0.2 ,and the bacterial solution was dipped into cotton swabs to evenly spread onto NA medium to make the bacterial plate containing the indicator strains.”

• Line 174: It is good to mention the preparation of fungal cake, but describe the type of agar that was used,and Line 178: Please mention which drug-sensitive paper was used.

Response:Thanks to the reviewer for asking this question, we have revised it in the manuscript:“a perforator with a diameter of 0.7cm was used to prepare bacterial cakes on the endophytic fungal colonies cultivated for 10 d. The bacterial cakes were placed on the bacterial plate containing the indicator strains in 3 groups of replicates per endophytic fungal strain with blanks. The fungus cake was prepared on the

endophytic fungal colonies with a 0.7 cm diameter punch, and placed on the fungus plate containing the indicator species, and 3 cakes were inoculated equidistant from each plate; 3 groups of replicates were made for each endophytic fungus, and the no antibiotics blank drug-sensitive paper(Shandong Best Microbial Technology Co., Ltd.) was used as the blank control, and the culture was incubated at a constant temperature of 28 °C, and the bacterial inhibitory activity was observed after 3 d. The results were summarized as follows,the size of the ring of inhibition was recorded and the average value was calculated."

Results (Lines 163-205):

• Line 222-229: It would be better to include a table of the Gram staining morphology results forbetter data presentation.

Response:Thanks to the reviewer's suggestion, we have taken your suggestion and have added the table to the document:"Table 4 Morphology and Gram staining of endophytic bacteria in the seeds of *Panax japonicus*."

• Line 242: Avoid the use of the abbreviation for "16S ribosomal DNA", write as "16S rDNA" for consistency.

Response:Thank you very much for reviewing our manuscript so carefully. We have made consistent revisions in the manuscript.

• Line 242-261: The method of identifying the endophytes is unclear. You should state the database that was used, such as NCBI (National Center for Biotechnology Information).Also, it would help to give more details regarding the process of phylogenetic tree construction.

Response:Thank you very much for the reviewer's careful suggestions, and we have taken the suggestions to supplement the manuscript:"BLAST search was performed at NCBI and a Utilization of the software MEGA11 phylogenetic tree was constructed (Fig.5), which revealed that the sequence similarity between endophytic fungus *Pj*Z1 and accession number JX827458.1 was 100% and identified as Fusarium tricinctum;*Pj*Z2 and accession number MT601889.1 had 100% sequence similarity and identified as Fusarium reticulatum."

• Line 276: Clarify that "Antagonistic experiments" are referring to the testing of

antibacterial activity. Also, clarify that the bacteria used in the antagonistic experiments were pathogenic.

Response:Thank you very much for the reviewer's suggestion. We have revised "Antagonistic experiments" to "Determination of bacteriostatic activity".

• Line 281-284: Describe the statistical significance of the differences observed and Line 298-307: The lack of activity should be presented in a table.

Response:Thank you very much for reminding us of this problem, we have added the table in the manuscript:Table 7 Inhibitory activity of endophytic fungal metabolites from the seeds of Panax japonicus and Table 8 Inhibitory activity of endophytic fungal metabolites from the seeds of Panax japonicus.

Discussion (Lines 308-376):

• Line 313: Clarify why the presence of Fusarium and Alternaria is important to discuss.

Response:Many thanks to the reviewer for raising this question, and we have made a new explanation in the manuscript:"Therefore, it is likely that the difficulty in seed germination of Panax japonicus is due to the action of these two endophytic fungi, and further research is needed to find antibiotics to inhibit the growth of this endophytic fungus in order to solve the problem of the difficulty in seed germination of Panax japonicus."

• Line 315: Is this significant? What do the non-inhibitory results suggest?•Line 320: Again, why is it important to note the dominant fungi were not found?Line 335: The authors should further discuss the importance of their finding in P. japonicus.?Line 337-344: Needs a better transition to the importance of Pseudomonas and Enterobacteriaceae.Line 361-365: Reiterate more clearly. The authors should briefly list the identified species, but also highlight the dominant species in the study,and Line 366-376: The conclusion is not comprehensive enough and should be revised to clearly state the main outcomes, limitations, and implications for future studies.

Response:"The or thogonal experiment screened out the optimal disinfection conditions of Panax japonicus seeds as 75% alcohol disinfection for 30s and 1% $HgCl_2$ disinfection for 9min, which had the lowest contamination rate of 3.33%.

Seven strains of endophytic fungi PjZ1, PjZ2, PjZ3, PjZ4, PjZ5, PjZ6, PjZ7 and three strains of endophytic bacteria PjX1, PjX2, PjX3 were isolated and screened from the seeds of Panax japonicus, and the strains of PjZ1, PjZ3, PjZ4, PjZ5 were identified as the fungi of the Fusarium genus, and the strains of PjZ2 and PjZ4 were identified as the fungi of the Fusarium genus. Fusarium reticulatum, PjZ6 is Fusarium sarcochroum, PjZ7 is Alternaria alternata; PjX1 and PjX3 are Enterobacteriaceae bacterium, and PjX2 is Pseudomonas aeruginosa. The results of inhibition experiments showed that only PjZ4 had inhibitory activity against E. coli, PjZ3, PjZ4, PjZ5, PjZ7 had inhibitory activity against Bacillus subtilis, and none of the 7 endophytic fungal strains had inhibitory activity against Staphylococcus aureus, and the metabolites of the 10 endophytic strains of endophytic fungi were antagonized with E. coli, Staphylococcus aureus, and Bacillus subtilis. The experiment showed that the metabolites of 10 strains of endophytic bacteria had no bacteriostatic activity against Escherichia coli, Staphylococcus aureus and Bacillus subtilis. After preliminary judgment, the isolated Fusarium spp. and Alternaria alternata. are not the dominant species in the growth and development process of Panax japonicus, and it can be inferred that the difficulty in seed germination of bamboo ginseng may be caused by the growth of these two genera of fungi inhibiting the synthesis of growth hormone by the seeds during the seed germination process. The isolated Enterobacteriaceae bacterium and Pseudomonas spp. are the dominant species in promoting plant growth and development, so these two genera of bacteria can be utilized in the cultivation process of Panax japonicus to promote its growth and development.The endophytes isolated in this study provide a theoretical basis for the screening of endophyte antagonist strains of Panax japonicus and for seed germination and disease control of Panax japonicus.”

Reviewer 1

For example, the paper's title is “Isolation, identification and antibacterial activity of endophytic bacteria from the seeds of Panax japonicus." However, the MS reports both bacterial and fungal endophytes. For instance, under the abstract, the authors write, “Seven endophytic fungi strains were isolated from the seeds of Panax

japonicus, named…………”

Response:Thanks to the reviewer for raising this question, we have revised the title into a more accurate text.

Again, thank you for giving us the opportunity to strengthen our manuscript with your valuable comments and queries. We have worked hard to incorporate your feedback and hope that these revisions persuade you to accept our submission.

Best regards.

Yours sincerely, Rui Jin

Corresponding author: Name: Lai Zhang

E-mail: 975575681@qq.com

January 14, 2025
