# Supplementary material for: Isolation, identification and antibacterial activity of endophytes from the seeds of Panax japonicus
Source: PLoS One. 2025 Oct 9;20(10):e0330436. doi: 10.1371/journal.pone.0330436 (PMC12510572; doi:10.1371/journal.pone.0330436)
Supplement: Attachment — Submitted filename: Response to Reviewers.docx [file pone.0330436.s005.docx]

Response to Reviewers

Dear editors and reviewers,

Thank you for your comments on the title of our manuscript 'Isolation, identification and antibacterial activity of endophyte from the seeds of *Panax japonicus* '. Your comments will help us to revise and improve the manuscript, and will also play an important guiding role in our future research in the Panax japonicus or other fields. We are very grateful to you for your comments and comments on the content of our manuscripts, especially for the questions we have pointed out in words. We have carefully studied these comments and tried to modify the manuscript, hoping to get your approval. Revisions are marked in red in the manuscript. The following is a response to your comments.

**Editor**

1.Please ensure that your manuscript meets PLOS ONE's style requirements, including thosefor file naming.

Response: Dear Editor, thank you for your reminder that we have modified the format and naming according to the journal requirements.

2.Please state what role the funders took in the study.

Response:Thanks to the editors for being so careful, we have clarified in the Cover letter: “The above four project funds provide financial support and project guidance for this research.”

3.We note that you have provided funding information that is currently declared in your Funding Statement. However, funding information should not appear in the Acknowledgments section or other areas of your manuscript. We will only publish funding information present in the Funding Statement section of the online submission form.Please remove any funding-related text from the manuscript and let us know how you would like to update your Funding Statement.

Response:Thanks for the editor's reminder and suggestion, we have made modifications, deleted the text related to funding from the manuscript, and included the funding information in the Cover letter.

4.When completing the data availability statement of the submission form, you indicated that you will make your data available on acceptance. We strongly recommend all authors decide on a data sharing plan before acceptance, as the process can be lengthy and hold up publication timelines. Please note that, though access restrictions are acceptable now, your entire data will need to be made freely accessible if your manuscript is accepted for publication. This policy applies to all data except where public deposition would breach compliance with the protocol approved by your research ethics board. If you are unable to adhere to our open data policy,please kindly revise your statement to explain your reasoning and we will seek the editor's input on an exemption. Please be assured that, once you have provided your new statement, the assessment of your exemption will not hold up the peer review process.

Response:Thanks for the editor's suggestion, we have started to prepare the work of data uploading and data sharing, and we promise to do our best to complete the work before the manuscript is accepted.

5.PLOS ONE now requires that authors provide the original uncropped and unadjusted images underlying all blot or gel results reported in a submission’s figures or Supporting Information Files.In your cover letter, please note whether your blot/gel image data are in Supporting Informationor posted at a public data repository, provide the repository URL if relevant, and provide specificdetails as to which raw blot/gel images, if any, are not available. Email us at plosone@plos.org if you have any questions.

Response:Thanks for the editors' valuable feedback and suggestions on the chart information. We have put the unadjusted chart into the file separately and explained it in the Cover letter: we will agree that blot/gel image data are in Supporting Information or posted at a public data repository.

6.Please remove your figures from within your manuscript file, leaving only the individual TIFF/EPS image files, uploaded separately ,and please include your tables as part of your main manuscript and remove the individual files.

Response:Thanks to the careful reminding of the editor, we have uploaded the single TIFF/EPS image file and deleted the chart from the manuscript, and the single table file has also been deleted.

**Reviewer 1**

Abstract (Lines 39-65):

• Line 40: Consider stating the specific aim of this study more clearly, such as: “The aim of this study was to isolate...”

Response:We agree with the reviewer on this point, and we have revised it in the manuscript: “ The purpose of this study is to isolate and identify endophytic bacteria from the seeds of *Panax japonicus*, finding a dominant strain that favors seed germination in *Panax japonicus* and to provide theoretical reference for further research on the effect of endophytic bacteria on seed germination. ”

• Line 48: It is confusing to read "Seven endophytic fungi strains were isolated" immediately followed by mentioning that "*Pj*Z1, *Pj*Z3, *Pj*Z4 and *Pj*Z5 were identified as Fusarium". This makes it appear that only these four were fungi and not all 7 strains. You should rephrase this to clarify

that ALL 7 are fungal.

Response:We thank the reviewer for making this important point, which we have elaborated in the manuscript: “Seven endophytic fungi strains were isolated from the seeds of *Panax japonicus*, named *Pj*Z1, *Pj*Z2, *Pj*Z3, *Pj*Z4, *Pj*Z5, *Pj*Z6, and *Pj*Z7, respectively. Four strains belonged to the genus *Fusarium tricinctum*, one to *Fusarium reticulatum*, one to *Fusarium sarcochroum*, and one to *Alternaria alternata”*

• Line 55: Instead of "Only PjZ4 had antibacterial activity", it may be more appropriate to say,"Among the isolates, only PjZ4 showed significant antibacterial activity...". This highlights that the other fungal isolates did have some inhibition on Bacillus subtilis, even if not significant.

Response:We thank the reviewer for pointing out a more appropriate statement for us, and we have readjusted it: “Among the endophytic fungal strains isolated, only PjZ4 showed significant bacteriostatic activity against Escherichia coli. ”

• Line 58: Conclusion: It is advised to have a stronger conclusion. For instance, the authors could summarize the main findings, including the genus level of the identified strains, and highlight the significance of their study for future research.

Response:Thanks to the reviewer for his valuable suggestions. In view of the suggestions, we have made a new explanation after careful consideration:“In this study, we investigated the method of bamboo ginseng seed disinfection, and the results showed that 75% alcohol disinfection for 30s and 1% HgCl_2_ disinfection for 9min resulted in the lowest contamination rate of 3.33%, in which the greatest influence on the seed disinfection effect was the concentration of HgCl_2_. Meanwhile, 11 endophyte species of *Panax japonicus* seeds were isolated and identified, *Fusarium spp*. and *Alternaria alternata*. were not the dominant species in the growth and development process of *Panax japonicus* while *Enterobacteriaceae* and *Pseudomonas sp.* were the dominant species in the promotion of plant growth and development, and provides a theoretical reference for further research on the biological functions and active substances of endophytic fungi in the seeds of *Panax japonicus*.”

• Line 63: The keywords could be improved by making them more specific. For example, replace "Antibacterial activity" with "Endophytic antibacterial activity".

Response:Thanks for your helpful suggestions, we have updated the keyword "Antibacterial activity" to "Endophytic antibacterial activity"

Introduction (Lines 66-81):

• Line 68: The phrase "also known as Disporopsis fuscopicta Hance" is an unnecessary detail here as it is not the same genus.

Response:We appreciate your reminder that we have removed the word from the manuscript.

• Line 70: Should add citations to support that "Pharmacological studies have shown that Panax japonicus..."

Response:We are grateful for your reminder that in the manuscript, our citation for this part reads [2-4]:[2]SHEN Peiyao, ZHANG Ye, LI Yuqin, YAN Jianye, WANG Yuanqing. Research progress on biological activity and quality control of Panacis Japonici Rhizoma[J]. Food and Machinery,2021,37(11):211-220. [3]GUO Zhe, FENG Zhitao, ZHANG Haoran, YAN Lan, LIANG Mingge, MEI Zhigang, CAI Sanjin. Progress of research on Panax japonicus and its preparation for the treatment of rheumatoid arthritis[J]. Chinese Materia Medica,2019,42(04):941-944. [4]LI Chunyan, ZHANG Jie, LI Jinping, et al. Research on Chemical Constituents and Biological Activities of Rhizoma of Panax japonicus[J]. Introduction to Traditional Chinese Medicine,2012,18(04):68-71.DOI:10.13862/j.cnki.cn43-1446/r.2012.04.050.

• Line 75: The statement regarding lack of reports on endophytic fungi/bacteria from seeds is good, but there needs to be something to indicate why this is valuable. For example, why are seeds and what importance is this to *P. japonicus*? And Line 77-80: The end of the introduction, there should be something to emphasize the biological functions of the bacteria isolated.

Response:We would like to thank the reviewer for pointing out this problem. The clarification of this problem will make the purpose of this paper more clear. Therefore, we have further elaborated this part in the manuscript:“Because of the high efficacy and high price of the rare wild Chinese herbal medicine, it is almost difficult to find wild resources of *Panax japonicus* in the wild, so the artificial propagation of Panax japonicus seeds is urgent.Therefore, the selection of *Panax japonicus* seeds, leaves and stems for seedling propagation is a better choice of explants. It is noteworthy that the seeds of *Panax japonicus* have the phenomenon of after-ripening, and the contamination rate is high during the germination process of histoculture, which makes the germination difficult. Therefore, we carried out further exploration. It has been shown that endophytes of medicinal plants are important for promoting the growth of medicinal plants, improving the quality of medicinal plants, and eliminating the barriers of continuous cultivation.”

Materials and Methods (Lines 82-162):

• Line 86: Provide the geographic coordinates or location details of the experimental site.

Response:We would like to thank the reviewer for suggesting that our detailed address is"Guizhou Innovation Center of Mountain Specialty and Efficient Agriculture" of Anshun College, Anshun City, Guizhou Province, China, has been presented in the manuscript.

• Line 90: The composition of NA (Nutrient Agar) should be described better. The recipe is given as "Peptone 10g, Beef Paste 3g, Sodium Chloride 5g, Agar 20g, distilled water 1000mL, pH:7.2." It would be better practice to say "Beef Extract" instead of "Beef Paste", as well as identify if peptone is tryptone or another form.

Response:We would like to thank the reviewers for their careful comments on this and we have corrected "Beef Extract" to" Beef paste".

• Line 103: Provide a citation or additional details to justify the use of this orthogonal testing method.

Response:Thank you very much for your professional advice and we have added relevant citations in this part of the manuscript:[8]ZHU Lili,ZHANG Yemeng,LI Wancai,et al. Comprehensive evaluation of the effects of different disinfectants on surface disinfection and germination of quinoa seeds[J]. Journal of Biology,2023,40(4):83-89. DOI:10.3969/j.issn.2095-1736.2023.04.083.

• Line 107-109: It is difficult to follow that section. Re-word and clarify that after disinfection, the seeds were plated to analyze contamination rate before proceeding with isolation.

Response:We thank the reviewers for their important comments on this, which we have rephrased in the manuscript:“Prior to the isolation of endophytes from the seeds of *Panax japonicus*, the seeds of *Panax japonicus* were sterilized under different conditions and the contamination rate under different sterilization conditions were analyzed.”

• Line 117: Clarify why the seed is cut in half before plating.

Response:Thank you very much for your professional advice and we have added relevant citations in this part of the manuscript:[9]ZHANG Yubai, WEN Xiangshui, WANG Yumeng, et al. Isolation and characterization of endophytic fungi from the seeds of Fructus schisandrae chinensis and their fungicidal properties[J]. Chinese Materia Medica,2020,43(5):1087-1091. DOI:10.13863/j.issn1001-4454.2020.05.008.

• Line 119: What is meant by "seed preservation" here? It is better to say for "preservation of the isolated strains"

Response:We thank the reviewers for their very meticulous comments, which we have reorganized: “After the growth of mycelium, pick the mycelium for repeated isolation and purification for 2-3 times for preservation of the isolated strains.”

• Line 127-136: The morphological observation section lacks clarity. How many samples were observed per isolate? Were observations done in replicates? More detail on how results were "recorded" should be included.

Response:Thanks to the reviewer's constructive comments on this, we have re-elaborated in the manuscript: “Pick the purified fungal hyphae and make them into clinical slices, observe and record (hyphae color, hyphae morphology, spore morphology, etc.) through an optical microscope, and refer to "Fungal Identification Manual".etc. for preliminary identification of fungi. (Three strains on each medium for each strain and three strains on each medium for observation and identification).”

• Line 137-139: The DNA extraction procedure needs to be described with more detail: What is the kit that was used? Also, it is better to use "according to the manufacture's instructions" rather than just stating "according to the instructions".

Response: Thanks to the reviewer for alerting us to this problem, which we have reflected in the manuscript:“Endophytic fungal DNA was extracted according to the instructions of the fungal DNA extraction kit (Solarbio), and endophytic bacterial DNA was extracted according to the instructions of the bacterial DNA extraction kit. Subsequently, PCR amplification was performed using the DNA of each endophytic bacterium as a template, respectively.”

• Line 144: The reaction volume and composition is confusing. It would be better to say "a 50uL reaction volume containing..." instead.

Response: Thanks to the reviewer's attention to the following details, we have corrected the statement:“Endophytic fungi were amplified by PCR using the universal primers of fungi, ITS1 (5'-TCCGTAGGTGAACCTGCGG-3') and ITS4 ( 5'-TCCTCCGCTTATTGATATGC-3') as primers, and the total volume of the PCR reaction system was 50μL, containing:4μL of DNA template, 2μL of each of ITS1 and ITS4, and 2×MasterMix 25μL, and add ddH2O 17μL.”

• Line 151: The statement about the primer name is inconsistent, the previous section mentioned primer ITS1 and ITS4 but now are described as "primers 27F (5'-AGAGTTTGATCCTGGCTCAG-3') and 1492R (5'-TACGGCTACCTTGTTACGAC".

Response: Thanks to the reviewer for raising this question. The inconsistent names of primers are due to the fact that the endophytic bacteria isolated in our paper include fungi and bacteria, and the common primers of the two bacteria are different, so the names and sequences of the two primers appear in the paper.

• Line 157: What percentage of agarose gel was used in gel electrophoresis?

Response:Thanks for the reviewer's attention to this issue: In the manuscript we mentioned that the percentage of agarose gel used was 1%.

• Line 166: Please add more detail about the selection of pure isolates. For instance, whether single colonies were used to prepare the pure isolates,and Line 167: What does “adjusted to OD600=0.2 in 0.85% saline”means? Also, how was the 0.2 OD reading achieved?

Response:Thanks to the reviewers, we have described the process more clearly in the manuscript and updated the text:“The pure indicator strains (Staphylococcus aureus, Bacillus subtilis, Escherichia coli) were picked and inoculated onto LB medium by plate streaking method for overnight cultivation; Pick single colonies on LB medium again for plate streaking overnight culture; pick single colonies to 0.85% saline to prepare the bacterial solution, adjust the concentration of the bacterial solution and measured by UV-visible spectrophotometer (UV-5500, Shanghai, China), until OD600 = 0.2 ,and the bacterial solution was dipped into cotton swabs to evenly spread onto NA medium to make the bacterial plate containing the indicator strains.”

• Line 174: It is good to mention the preparation of fungal cake, but describe the type of agar that was used,and Line 178: Please mention which drug-sensitive paper was used.

Response:Thanks to the reviewer for asking this question, we have revised it in the manuscript:“a perforator with a diameter of 0.7cm was used to prepare bacterial cakes on the endophytic fungal colonies cultivated for 10 d. The bacterial cakes were placed on the bacterial plate containing the indicator strains in 3 groups of replicates per endophytic fungal strain with blanks. The fungus cake was prepared on the endophytic fungal colonies with a 0.7 cm diameter punch, and placed on the fungus plate containing the indicator species, and 3 cakes were inoculated equidistant from each plate; 3 groups of replicates were made for each endophytic fungus, and the no antibiotics blank drug-sensitive paper(Shandong Best Microbial Technology Co., Ltd.) was used as the blank control, and the culture was incubated at a constant temperature of 28 ℃, and the bacterial inhibitory activity was observed after 3 d. The results were summarized as follows,the size of the ring of inhibition was recorded and the average value was calculated.”

Results (Lines 163-205):

• Line 222-229: It would be better to include a table of the Gram staining morphology results forbetter data presentation.

Response:Thanks to the reviewer's suggestion, we have taken your suggestion and have added the table to the document:"Table 4 Morphology and Gram staining of endophytic bacteria in the seeds of *Panax japonicus*.”

• Line 242: Avoid the use of the abbreviation for "16S ribosomal DNA", write as "16S rDNA" for consistency.

Response:Thank you very much for reviewing our manuscript so carefully. We have made consistent revisions in the manuscript.

• Line 242-261: The method of identifying the endophytes is unclear. You should state the database that was used, such as NCBI (National Center for Biotechnology Information).Also, it would help to give more details regarding the process of phylogenetic tree construction.

Response:Thank you very much for the reviewer's careful suggestions, and we have taken the suggestions to supplement the manuscript:“BLAST search was performed at NCBI and a Utilization of the software MEGA11 phylogenetic tree was constructed (Fig.5), which revealed that the sequence similarity between endophytic fungus *Pj*Z1 and accession number JX827458.1 was 100% and identified as Fusarium tricinctum;*Pj*Z2 and accession number MT601889.1 had 100% sequence similarity and identified as Fusarium reticulatum.”

• Line 276: Clarify that “Antagonistic experiments” are referring to the testing of antibacterial activity. Also, clarify that the bacteria used in the antagonistic experiments were pathogenic.

Response:Thank you very much for the reviewer's suggestion. We have revised "Antagonistic experiments" to "Determination of bacteriostatic activity".

• Line 281-284: Describe the statistical significance of the differences observed and Line 298-307: The lack of activity should be presented in a table.

Response:Thank you very much for reminding us of this problem, we have added the table in the manuscript:Table 7 Inhibitory activity of endophytic fungal metabolites from the seeds of Panax japonicus and Table 8 Inhibitory activity of endophytic fungal metabolites from the seeds of Panax japonicus.

Discussion (Lines 308-376):

• Line 313: Clarify why the presence of Fusarium and Alternaria is important to discuss.

Response:Many thanks to the reviewer for raising this question, and we have made a new explanation in the manuscript:“Therefore, it is likely that the difficulty in seed germination of Panax japonicus is due to the action of these two endophytic fungi, and further research is needed to find antibiotics to inhibit the growth of this endophytic fungus in order to solve the problem of the difficulty in seed germination of Panax japonicus.”

• Line 315: Is this significant? What do the non-inhibitory results suggest?•Line 320: Again, why is it important to note the dominant fungi were not found?Line 335: The authors should further discuss the importance of their finding in P. japonicus.?Line 337-344: Needs a better transition to the importance of Pseudomonas and Enterobacteriaceae.Line 361-365: Reiterate more clearly. The authors should briefly list the identified species, but also highlight the dominant species in the study,and Line 366-376: The conclusion is not comprehensive enough and should be revised to clearly state the main outcomes, limitations, and implications for future studies.

Response:“The or thogonal experiment screened out the optimal disinfection conditions of Panax japonicus seeds as 75% alcohol disinfection for 30s and 1% HgCl2 disinfection for 9min, which had the lowest contamination rate of 3.33%. Seven strains of endophytic fungi PjZ1, PjZ2, PjZ3, PjZ4, PjZ5, PjZ6, PjZ7 and three strains of endophytic bacteria PjX1, PjX2, PjX3 were isolated and screened from the seeds of Panax japonicus, and the strains of PjZ1, PjZ3, PjZ4, PjZ5 were identified as the fungi of the Fusarium genus, and the strains of PjZ2 and PjZ4 were identified as the fungi of the Fusarium genus. Fusarium reticulatum, PjZ6 is Fusarium sarcochroum, PjZ7 is Alternaria alternata; PjX1 and PjX3 are Enterobacteriaceae bacterium, and PjX2 is Pseudomonas aeruginosa. The results of inhibition experiments showed that only PjZ4 had inhibitory activity against E. coli, PjZ3, PjZ4, PjZ5, PjZ7 had inhibitory activity against Bacillus subtilis, and none of the 7 endophytic fungal strains had inhibitory activity against Staphylococcus aureus, and the metabolites of the 10 endophytic strains of endophytic fungi were antagonized with E. coli, Staphylococcus aureus, and Bacillus subtilis. The experiment showed that the metabolites of 10 strains of endophytic bacteria had no bacteriostatic activity against Escherichia coli, Staphylococcus aureus and Bacillus subtilis. After preliminary judgment, the isolated Fusarium spp. and Alternaria alternata. are not the dominant species in the growth and development process of Panax japonicus, and it can be inferred that the difficulty in seed germination of bamboo ginseng may be caused by the growth of these two genera of fungi inhibiting the synthesis of growth hormone by the seeds during the seed germination process. The isolated Enterobacteriaceae bacterium and Pseudomonas spp. are the dominant species in promoting plant growth and development, so these two genera of bacteria can be utilized in the cultivation process of Panax japonicus to promote its growth and development.The endophytes isolated in this study provide a theoretical basis for the screening of endophyte antagonist strains of Panax japonicus and for seed germination and disease control of Panax japonicus.”

**Reviewer 1**

For example, the paper's title is “Isolation, identification and antibacterial activity of endophytic bacteria from the seeds of Panax japonicus." However, the MS reports both bacterial and fungal endophytes. For instance, under the abstract, the authors write, “Seven endophytic fungi strains were isolated from the seeds of Panax japonicus, named…………”

Response:Thanks to the reviewer for raising this question, we have revised the title into a more accurate text.

Again, thank you for giving us the opportunity to strengthen our manuscript with your valuable comments and queries. We have worked hard to incorporate your feedback and hope that these revisions persuade you to accept our submission.

Best regards.

Yours sincerely, Rui Jin

Corresponding author: Name: Lai Zhang

E-mail: [975575681@qq.com](mailto:975575681@qq.com)

January 14, 2025
