# Supplementary material for: Isolation, identification and antibacterial activity of endophytes from the seeds of Panax japonicus
Source: PLoS One. 2025 Oct 9;20(10):e0330436. doi: 10.1371/journal.pone.0330436 (PMC12510572; doi:10.1371/journal.pone.0330436)
Supplement: Attachment — Submitted filename: Response_to_Reviewers_auresp_2.docx [file pone.0330436.s006.docx]

Response to Reviewers

Dear editors and reviewers,

Thank you for your comments on the title of our manuscript 'Isolation, identification and antibacterial activity of endophyte from the seeds of Panax japonicus '. Your comments will help us to revise and improve the manuscript, and will also play an important guiding role in our future research in the Panax japonicus or other fields. We are very grateful to you for your comments and comments on the content of our manuscripts, especially for the questions we have pointed out in words. We have carefully studied these comments and tried to modify the manuscript, hoping to get your approval. Revisions are marked in red in the manuscript. The following is a response to your comments.

**Reviewer 3**

1. The title doesn't specify what group of microbial endophytes that the researchers are focusing on, but the introductory part of the abstract only mentions "endophytic bacteria" as the purpose of this study (L.26-29). However, the result part of the abstract suddenly focuses on "Seven endophytic fung[al] strains" L.34 and "Three strains of endophytic bacteria" L.38 at which sum up to be "11 endophyte species" L.50 in the conclusion part of the abstract. This bring up the question of where the number 11 come from. This is on top of the inconsistent issue with the correct way to write all scientific names both in the abstract and in the rest of the manuscript.

Response:We thank the reviewer for making this important point，After rechecking, we have corrected the errors in the manuscript caused by our carelessness. A total of 10 endophyte are mentioned in the article, and we have also checked and corrected all scientific names.

2. L.59 I[n]troduction
   The beginning of the introduction is a bit confusing and should start with "The chemical composition of dried rhizome of Panax japonicus C.A. Mey. mainly includes ..." For the rest of the introduction, any scientific name that has already been written in full previously, you should abbreviate the genus name for the subsequent use in the manuscript.

Response:We thank the reviewer for pointing out a more appropriate statement for us，and we have readjusted it:“*Panax japonicus* C.A. Meyer. is a famous folk medicine of the Tujia ethnic group, and is regarded as the “king of herbs’ by the Tujia ethnic group of Enshi Hubei. ”

3. Materials and methods:
    Culture medium section is very confusing. Components of some media were described in details while some appears to be side-of-the-bottle instruction on how to prepare them while many only have the names of the media mentioned without any details. Also, one medium that was mentioned in the "Disinfection" sub-section was not included L.114.

Response:Thanks for your helpful suggestions, we have updated ：“Fungi isolation medium: Potato Dextrose Agar Medium (PDA solid medium) 40.1g, distilled water 1000mL, pH:5.8. Bacterial isolation medium: Beef Extract Peptone Medium (NA solid medium): Peptone 10g, Beef Extract 3g, Sodium Chloride 5g, Agar 20g, distilled water 1000mL, pH:7.2. Experimental Medium for Fungi Inhibition Nutrient broth (NB liquid medium): peptone 10g, Beef Extract 3g, sodium chloride 5g, distilled water 1000mL, pH: 7.2, Beef Extract Peptone Medium (NA solid medium). Fungi fermentation medium: PDB liquid medium, Beef Extract Peptone Medium (NA solid medium), Nutrient Broth (NB liquid medium). PDA, NA and NB medium are prepared as above.Bacterial fermentation medium: LB solid medium: LB dry powder 25g, distilled water 1000mL, Agar 15g pH:7.0; LB liquid medium: LB dry powder 25g, distilled water 1000mL, pH:7.0; MH medium: Mueller-Hinton Agar 38g, distilled water 1000mL, pH:7.3”

4. The sub-section on "Screening of seed disinfection conditions" is even more confusing because you described the "disinfection time of 6min, 12min, 18min" L.105 which make me wonder where the "9min" in the rest of your manuscript comes from.
    Response:We are grateful for your reminder that in the manuscript,I sincerely apologise for the confusion caused by our negligence.We used time intervals of 3 minutes, 6 minutes, and 18 minutes, and have corrected this in the manuscript.
5. L.126 "the same as 1.6 Seed Disinfection Method" What does 1.6 refer to?L.129 is very confusing. What is the "and line the purification, purify 2-3 times to keep the seeds.L.135-136 (Three strains on each medium for each strain and three strains on each medium for observation and identification).

Response:We would like to thank the reviewer for pointing out this problem.This is an error that we left behind when revising the first draft, and we have noted it in the manuscript：“Take fresh seeds of *P. japonicus* and rinse them under running water, then rinse them 1-3 times with sterile water, same as above for seed disinfection, peel the seeds and cut them in half, place them on NA medium with the cut side facing down, and place them in the incubator at 28℃ for cultivation. After the growth of colonies, the strains were purified using the streak plate method, the strains were purified 2-3 times and then preserved for isolated line purification.”

6. Molecular biology identification:
   L.142 mentioned that "Endophytic fungal DNA was extracted" but then on the same paragraph L.146 why "DNA of each endophytic bacterium as a template"? Also, the PCR conditions were described inconsistently regarding the volume of each components and there was no mention of concentration of the primers used and no reference to them.
    Response:Thanks to the reviewer for alerting us to this problem,We have corrected the description in the manuscript：“extracted DNA was used as a template for PCR amplification respectively.”and “The primer concentrations mentioned above were all 10 μmol.”
7. L.178-185 were very confusing.L.187 L.201 How did you measure the size of the ring of inhibition? Area? Circumference? Diameter?L.210-211 A bit unclear writing.

Response: Thanks to the reviewer for raising this question.We measured the diameter of the inhibition ring,We have corrected the original statement in the manuscript:“a perforator with a diameter of 0.7cm was used to prepare fungi chunks were prepared on the endophytic fungi colonies with a 0.7 cm diameter punch, and placed on the fungi plate containing the indicator species, and 3 chunks were inoculated equidistant in each plate”.

8. L.215 What is 1.9.2?There are no mention to Fig.1 and Fig 2 in text.

Response:Thank you very much for reviewing our manuscript so carefully.We have corrected the original statement in the manuscript:“Determination of bacteriostatic activity of endophytic bacterial metabolites was the same as above for the determination of bacteriostatic properties of endophytic fungi metabolites.”and “The external morphology and micromorphology of fungi and bacterial are shown in Fig.1 and Fig.2.”

9. For the metabolites perhaps there is no need to tables 7 and simply report as no effect. Also, is table 8 the exact of table 7?
    Response:Thank you very much for reminding us of this problem,We have corrected table 8 in the manuscript.
10. L.382 What is "salt tolerance after ping"?The Conclusion is also very confusing with regard to "dominant species" and here we found "10 endophytic strains of endophytic fungi" L.407 and "10 strains of endophytic bacteria" L.409. Moreover, your study did not have any experiment on the effect of isolated microbial endophytes on the growth and development process of Panax japonicus at all and thus appears to be over claiming.

Response:Thank you very much for reminding us of this proble,In the manuscript, we replaced the concept of “dominant species “with “growth-promoting endophyte”,we have corrected the original statement in the manuscript:“Some studies have shown that *Enterobacteriaceae* has a better effect in promoting plant growth, such as Zhao isolated endophytic *Enterobacteriaceae* from the *Paspalum vaginatum* as a material to explore the germination of *Cynodon daclylon* seeds and salt tolerance after growing into a lawn, the results of the seed germination rate and salt tolerance after growing into a lawn has been improved.”This study preliminarily performed the isolation of endophytes and investigation of their antibacterial activities. In the future, we will focus on applying the isolated endophytes to the seed germination of *Panax japonicus*, with the aim of providing convenience for the production of *Panax japonicus*.

**Reviewer 4**

1.The authors have not clearly or justifiably defined the purpose, context, and rationale of their study in a way that a broader audience, even an interdisciplinary scientific audience,can easily understand. They jump into procedural detail without first explaining why the work matters in a straightforward way. Ambiguities and missing explanation raise these question in introduction section: Why are the seeds being germinated in a lab? What problem is being solved? (The manuscript mentions contamination, but doesn't clearly connect it to seed failure, dormancy, and germination goals). Who benefits from this research? Is it for agricultural production, conservation, medicinal compound extraction? This is only vaguely implied. Why are E. coli, S. aureus, and B. subtilis used in tests? Not explained in ecological or applied terms.
 Response:We thank the reviewers for their important comments on this,we have carefully revised the article based on the issues you raised.in the manuscript, we mentioned:“Because of the high efficacy and high price of the rare wild Chinese herbal medicine, it is almost difficult to find wild resources of *P. japonicus* in the wild, so the artificial propagation of *P. japonicus* seeds is urgent.”The special growth environment of *P. japonicus* necessitates the preliminary seed germination in laboratories to create the specific conditions required for its cultivation,and this study aims to provide insights into the relevant issues encountered in the cultivation and production of *P. japonicus*.Additionally, since the citations in the manuscript involved determining the antibacterial effects of these three indicator bacteria against seed endophytes, we adopted this method to conduct a preliminary exploration of the endophytes in *P. japonicus*.We have also been inspired by your reminder, and we will strive to do better in our future research.

2.The use of HgCl_2_ poses health and environmental risks. Recent protocols favor bleach (NaOCl) or H_2_O_2_ alternatives with comparable efficacy . Justify choice of HgCl₂ over safer agents, or test alternatives.
 Response:Thanks for your helpful suggestions.In the early stages, we used NaOCl for disinfection; however, it damaged the seed coat and negatively impacted seed germination. Therefore, we used HgCl_2_ for the experiment. In the future, we will explore greener and safer reagents for use in our research based on your suggestions.

3.Antibacterial activity was only measured as inhibition zones from agar plugs and disc diffusion. For rigorous comparison,why minimal inhibitory concentrations (MICs) or broth microdilution assays were not performed?

Response:We thank the reviewer for making this important point.Before using this bacterial solution concentration, we conducted concentration testing experiments. The results showed that the best experimental results were obtained at DO_600_=0.2, and we ultimately decided to use this concentration for our research.

4.Have you considered testing endophyte re-inoculation on seed germination or seedling growth? Such functional assays are critical to demonstrate PGP potential and vave you deposited all sequence data in GenBank with accession numbers?

Response:We are grateful for your reminder that in the manuscript,This study only conducted preliminary endophyte isolation and antibacterial experiments, without functional verification. We will proceed with this in the next step. In addition, we are currently uploading all sequence data .

5.Have you considered testing endophyte re-inoculation on seed germination or seedling growth? Such functional assays are critical to demonstrate PGP potential and vave you deposited all sequence data in GenBank with accession numbers?Instead of reporting what was done, the authors write Methods section as if they are giving instructions, like a lab manual.These kinds of descriptions r didactic, rather than declarative and scientific.For a research article, methods should be written in the past tense, reporting what was actually done, rather than describing steps as if in a lab manual.The manuscript STILL REQUIRES careful revision for grammatical accuracy, sentence structure, and scientific terminology. Clearer sentence construction and restructuring of ambiguous or repetitive descriptions are recommended to improve clarity and precision. PLEASE go through the manuscript and check for grammatical/structural errors.

Response:We thank the reviewer for making this important point,which we have made every effort to correct the tense and grammar based on your reminder, and we hope that this will resolve the issue.

6.I CONCLUDE THESE MAJOR LIMITATION IN YOUR WORK: (Should be acknowledged in relevant section): The antibacterial activity results are modest,only one fungus showed weak inhibition, and none of the metabolites did. No functional testing was done (yet) to prove that these endophytes help or harm seed germination directly. It is more of a foundational study, setting the stage for future work (e.g., applying isolated strains to seeds and testing growth impact).

Response:Thanks to the reviewer's suggestion,we have taken your suggestion and have added the manuscript:“The antibacterial activity results are modest,for fungus showed weak inhibition, and none of the metabolites did. No functional testing was done to prove that these endophytes help or harm seed germination directly. It is more of a foundational study, setting the stage for future work.Next, we will further apply the isolated endophytic bacteria to seeds and test their effect on seed germination.”

Again, thank you for giving us the opportunity to strengthen our manuscript with your valuable comments and queries. We have worked hard to incorporate your feedback and hope that these revisions persuade you to accept our submission.

Best regards.

Yours sincerely, Rui Jin

Corresponding author: Name: Lai Zhang

E-mail:[975575681@qq.com](mailto:975575681@qq.com)

June 3, 2025
